# Caffeic Acid-Driven Green Synthesis of Rhenium Nanoparticles Embedded in Self-Templating Double-Shelled ZnMn_2_O_4_ Hollow Microspheres for Ultrasensitive Epinephrine Detection in Biofluids

**DOI:** 10.1021/acssensors.5c03023

**Published:** 2025-11-27

**Authors:** Rajalakshmi Sakthivel, Bo-Yuan Chen, Subbiramaniyan Kubendhiran, Lu-Yin Lin, Sayee Kannan Ramaraj, Yu-Chien Lin, Xinke Liu, Cihun-Siyong Gong, Ren-Jei Chung

**Affiliations:** † Department of Chemical Engineering and Biotechnology, 427107 National Taipei University of Technology (Taipei Tech), Taipei 10608, Taiwan; ‡ PG& Research Department of Chemistry, 29967 Thiagarajar College, Madurai 625009, Tamil Nadu, India; § School of Materials Science and Engineering, 54761 Nanyang Technological University, Singapore 639798, Singapore; ∥ BIOBOND LTD, Corsham, Wiltshire SN13 0B, U.K.; ⊥ College of Materials Science and Engineering, Chinese Engineering and Research Institute of Microelectronics, 47890 Shenzhen University, Shenzhen 518060, China; # Department of Electrical and Computer Engineering, National University of Singapore, Singapore 117583, Singapore; ∇ Department of Electrical Engineering, 34911 National Central University, Zhongli, Taoyuan 320317, Taiwan; ○ High-value Biomaterials Research and Commercialization Center, National Taipei University of Technology (Taipei Tech), Taipei 10608, Taiwan

**Keywords:** green synthesis, rhenium nanoparticles, double-shell ZnMn_2_O_4_, epinephrine sensing, biofluids

## Abstract

In the present work, we report an electrochemical sensor based on rhenium (Re) nanoparticles embedded within a double-shelled ZnMn_2_O_4_ hollow microsphere (Re@ZnMn_2_O_4_) for ultrasensitive detection of epinephrine (EP) in biofluids. The Re@ZnMn_2_O_4_ material was synthesized via a coprecipitation and annealing route, followed by a green, caffeic acid (CA)-assisted chemical reduction. Structural and morphological analyses, including spectrophotometry, confirmed the high purity, crystallinity, and integrity of the material. Electrochemical performance was evaluated using voltammetry and impedance spectroscopy. The Re@ZnMn_2_O_4_-modified electrode exhibited superior electrochemical activity attributed to its high conductivity, large surface area, abundant active sites, and efficient charge transfer enabled by the hollow architecture. EP oxidation followed a diffusion-controlled 2e^–^/2H^+^ transfer mechanism. The sensor demonstrated a broad linear detection range (0.5–1951.3 μM), a low detection limit (0.21 μM), and good sensitivity (0.282 μA μM^–1^ cm^–2^). Furthermore, it showed remarkable reproducibility, long-term stability, and strong resistance to common interferents. Its practical potential was validated by accurate EP quantification in human serum and urine, highlighting its applicability in clinical diagnostics and biomedical monitoring.

## Graphical abstract

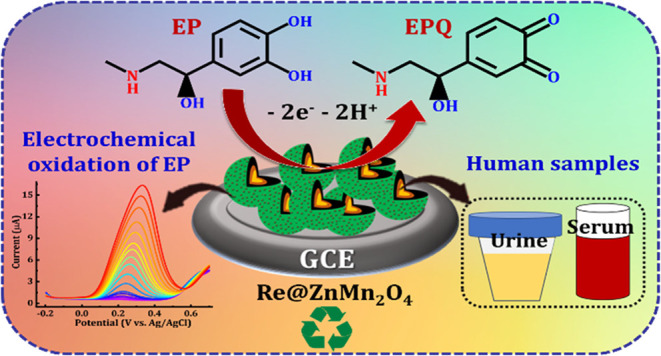

Epinephrine ((4-[(1R)-1-hydroxy-2-(methylamino)­ethyl]­benzene-1,2-diol), EP), a vital neurotransmitter and hormone secreted by the adrenal glands, plays a pivotal role in regulating cardiovascular function, metabolism, and the body’s response to stress.^1^ Alterations in EP levels are implicated in various pathophysiological conditions, including adrenal tumors and hypoglycemic unawareness. Additionally, EP serves as a critical therapeutic agent in emergency medicine, notably in the management of anaphylactic reactions, cardiac arrest, and septic shock.^2^ Given the critical role of EP levels in various physiological processes, there is a pressing need for the development of precise sensors capable of accurately quantifying EP concentrations in biofluids.^3^ Under normal conditions, plasma EP concentrations in adults typically remain below 10 ng/L. However, during physical activity, these levels can increase up to 10-fold, and during periods of stress, they may rise up to 50-fold or more. This significant elevation is attributed to the activation of the sympathetic-adrenal system, which stimulates the adrenal medulla to release EP into the bloodstream.^4,5^ Hence, a pressing need for the development of precise sensors capable of accurately quantifying EP concentrations in biofluids.^6^ To date, various analytical techniques applied for EP sensing include liquid chromatography,^7^ spectrophotometry,^8^ flow injection analysis,^9^ capillary electrophoresis,^10^ and fluorimetry.^11^ Nonetheless, these techniques often necessitate intricate and time-intensive sample preparation procedures, leading to increased operational costs and extended analysis times.^12^ Consequently, bio/chemical sensors have gained significant attention in modern analytical chemistry due to their enhanced sensitivity, specificity, and potential for real-time, on-site analysis.^13^ Electrochemical sensors have garnered attention due to their simple fabrication, fast response times, high precision, low cost, compact size, and compatibility,^14^ making them an effective alternative to complex analytical techniques.^15–18^ Unmodified electrodes often exhibit limited electron transfer rates and poor conductivity. Therefore, modifying them with nanostructured or alternative materials is essential to enhance their electrochemical performance.^19^

Researchers are increasingly focusing on semiconducting transition metal oxide materials due to their enhanced electrical conductivity, catalytic activity, and electrochemical performance, making them promising candidates for various applications.^20^ The AB_2_O_4_ spinel structures are mixed-valence oxides that exhibit more complex redox processes compared to single-component oxides.^21^ Significantly, spinel zinc manganate (ZnMn_2_O_4_), characterized by divalent zinc occupying tetrahedral sites and trivalent manganese occupying octahedral sites, exhibits enhanced redox properties. This material is notably eco-friendly, inexpensive, and nontoxic, making it a promising candidate for various applications.^22,23^ Besides, ZnMn_2_O_4_ exhibits superior structural flexibility and enhanced electrical conductivity, owing to the high mobility of surface electrons.^24^ ZnMn_2_O_4_ is commonly synthesized using coprecipitation methods^25^ or sol–gel methods,^26^ followed by thermal treatment to achieve the desired phase and crystallinity. Mukherjee et al. synthesized graphite-sheathed ZnMn_2_O_4_ microspheres via a hydrothermal method for the electrochemical detection of As­(III). This approach enhances the charge transport properties of ZnMn_2_O_4_, making it a promising material for electrochemical sensing platforms.^24^ Besides, Li et al. reported using ZnMn_2_O_4_ microspheres by the solvothermal method, and it has excellent electrocatalytic activity for H_2_O_2_ sensing.^27^ As a consequence, we have synthesized double-shell ZnMn_2_O_4_ microspheres using the coprecipitation approach.

Introducing transition metals into sensing materials effectively enhances the sensitivity and selectivity of electrochemical sensors while also speeding up their response and recovery times.^28^ Rhenium (Re) stands out for its unique combination of chemical, electronic, and mechanical properties. Its strong resistance to deactivation by sulfur, nitrogen, and phosphorus makes it suitable for use in high-performance areas like aerospace, electronics, cancer treatment, and electrocatalysis.^29^ Re-based materials are considered among the most promising candidates for versatile catalytic applications. Beyond their use in pure metallic fles (Re NPs) are often employed as catalysts when supported on various substrates, namely polymers,^30^ carbon materials,^31^ DNA scaffolds,^32^ and γ-Al_2_O_3_,^33^ among others.^34^ Incorporating Re NPs into suitable solid supports, as in catalysts,^35^ enhances not only the support function but also the metal–support interface chemistry, electronic structure, and catalytic efficiency. Key features, such as high surface area, fast electron transfer, pore volume, synergistic effects, and enhanced conductivity, primarily drive the electrochemical sensing performance.^36^ Re NPs initially form in metallic states but tend to oxidize spontaneously to stabilize their surface. This oxidation, however, can be managed by the synthesis conditions, choice of capping agents, and control of particle size.^32^ Traditional synthesis often relies on many chemicals and causes environmental pollution, while greener methods are attracting interest but are still not widely studied.^37,38^ In this work, Re NPs sol was synthesized by a green approach using caffeic acid (CA), a plant-based antioxidant and strong reducing agent, with ammonium perrhenate as the oxidant and CTAB as a shape-directing agent.^39^ The resulting Re@CTA­(aq) sol served as a sacrificial seed template for producing Re NPs.

Inspired by the above consideration, in this work, we developed a Re@ZnMn_2_O_4_ modified electrode to detect EP in biofluids. The double shell ZnMn_2_O_4_ hollow spheres were synthesized via coprecipitation followed by annealing, while the Re@ZnMn_2_O_4_ was synthesized using chemical reduction using CA. The resulting Re@ZnMn_2_O_4_ had superior electrochemical characteristics toward EP sensing. These characteristics improve detection efficiency, enable precise target recognition, and ensure stable performance. The developed sensor provides a broad detection range and a low detection limit, making it suitable for real-time clinical monitoring.

## Experimental Section

Comprehensive details regarding the chemicals, reagents, and characterization techniques can be found in Sections S1 and S2 of the Supporting Information.

### Synthesis of Double-Shell ZnMn_2_O_4_ Hollow Spheres

Slight modification to the prior report,^40^ to synthesize ZnMn_2_O_4_, 0.2875 g of zinc sulfate heptahydrate (ZnSO_4_·7H_2_O), 1.226 g of manganese sulfate tetrahydrate (MnSO_4_·4H_2_O), 1.725 g of sodium tartrate dihydrate (Na_2_C_4_H_4_O_6_·2H_2_O), and 2.803 g of hexamethylenetetramine (C_6_H_12_N_4_) were dissolved in 50 mL of deionized water and stirred until fully dissolved. Na_2_C_4_H_4_O_6_·2H_2_O acts as a chelating agent, binding to Zn^2+^ and Mn^2+^ ions, stabilizing them in solution and preventing premature precipitation (step 1). The resulting solution was heated in a water bath at 95 °C for 1.5 h, during which C_6_H_12_N_4_ gradually decomposed (step 2), releasing OH^–^ ions that reacted with the metal (Zn^2+^ and Mn^2+^) ions to form a hydroxide (OH^–^) precursor (step 3). Following the reaction, the solution was allowed to cool naturally to room temperature and was left undisturbed for 15 h. The resulting suspension was vacuum filtered to collect the white precipitate, which was then thoroughly washed with deionized water five times to eliminate any remaining ions. The purified precipitate was dried in a vacuum oven at 60 °C for 12 h. Finally, the dried hydroxide powder was calcined in a furnace by heating it to 550 °C at a rate of 2 °C per minute, maintained for 2 h, and then allowed to cool to room temperature, resulting in the formation of spinel ZnMn_2_O_4_ (step 4).

Step 1: (Dissolution and complex formation)

1 $${\mathrm{ZnSO}}_{4}\cdot 7H_{2}O\to {\mathrm{Zn}}^{2+}+{{\mathrm{SO}}_{4}}^{2-}+7H_{2}O$$

2 $${\mathrm{MnSO}}_{4}\cdot 4H_{2}O\to {\mathrm{Mn}}^{2+}+{{\mathrm{SO}}_{4}}^{2-}+4H_{2}O$$

3 $${\mathrm{Na}}_{2}C_{4}H_{4}O_{6}\cdot 2H_{2}O\to 2{\mathrm{Na}}^{+}+C_{4}H_{4}{O_{6}}^{2-}+2H_{2}O$$

Step 2: (Thermal decomposition of HMTA)

4 $$C_{6}H_{12}N_{4}+6H_{2}O\to 6\mathrm{HCHO}+4{\mathrm{NH}}_{3}$$

and

5 $${\mathrm{NH}}_{3}+H_{2}O⇌{{\mathrm{NH}}_{4}}^{+}+{\mathrm{OH}}^{-}$$

Step 3: (Precipitation of metal hydroxides)

6 $${\mathrm{Zn}}^{2+}+2{\mathrm{Mn}}^{2+}+{\mathrm{OH}}^{-}\left(\mathrm{from HMTA}\right)\to \mathrm{Zn}{\left(\mathrm{OH}\right)}_{2}+2\mathrm{Mn}{\left(\mathrm{OH}\right)}_{2}$$

Step 4: Final step (calcination)

7 $$\mathrm{Zn}{\left(\mathrm{OH}\right)}_{2}+2\mathrm{Mn}{\left(\mathrm{OH}\right)}_{2}\to {\mathrm{ZnMn}}_{2}O_{4}\left(\mathrm{spinel}\right)+H_{2}O↑$$

### Synthesis of Re@ZnMn_2_O_4_

The Re@ZnMn_2_O_4_ material was prepared through an environmentally friendly synthesis route utilizing caffeic acid (CA) as a natural reducing agent. To begin, 100 mg of ZnMn_2_O_4_ powder was suspended in 22 mL of deionized water and stirred magnetically for 30 min to achieve uniform dispersion. In parallel, 26.82 mg of ammonium perrhenate (NH_4_ReO_4_) was dissolved in 8 mL of deionized water, while 36.44 mg of CTAB was separately dissolved in 5 mL of the same solvent, both under magnetic stirring until complete dissolution. The NH_4_ReO_4_ solution was then gradually introduced into the ZnMn_2_O_4_ dispersion and stirred for 15 min. This was followed by the addition of the CTAB solution, with mixing continued for an additional 15 min to facilitate interaction among components. Afterward, 9.00 mg of CA was added to the combined solution, and the mixture was stirred at room temperature for 6 h to allow in situ reduction and deposition of Re nanoparticles on the ZnMn_2_O_4_ surface.

A green chemical reduction approach was employed to synthesize elemental Re NPs, using the reaction between perrhenate ions (ReO_4_^–^) and CA as the reducing agent. The process was carried out at ambient temperature, with CTAB serving as a stabilizer to control NPs growth and prevent agglomeration. The proposed formation mechanism is illustrated below. In the initial step, CTAB dissociates into CTA^+^ and Br^–^ ions. Subsequently, the ReO_4_^–^ ion electrostatically associates with the CTA^+^ cation, leading to the formation of a CTA@ReO_4_ complex.^39^

8 $$\mathrm{Step}1:\;{\mathrm{CTAB}}_{\left(\mathrm{aq}\right)}⇌{\mathrm{CTA}}_{\left(\mathrm{aq}\right)}^{+}+{\mathrm{Br}}^{-}$$

9 $$\mathrm{Step}\;2:{\mathrm{CTA}}_{\left(\mathrm{aq}\right)}^{+}+{\mathrm{ReO}}_{4\left(\mathrm{aq}\right)}^{-}⇌\mathrm{CTA}@{\mathrm{ReO}}_{4\left(\mathrm{aq}\right)}$$

Upon introducing CA, ReO_4_^–^ undergoes a green redox reaction and is reduced to elemental Re^0^, while CA is simultaneously oxidized to its corresponding quinone form.

10 Step3:2CTA@ReO4(aq)+CA(aq)→2Re0@CTA(aq)+oxidized CA(quinone)(aq)+4H2O

Finally, during the reaction, ZnMn_2_O_4_ microspheres act as a supporting substrate, facilitating the in situ formation and deposition of Re^0^ NPs on their surface to yield the Re^0^@ZnMn_2_O_4_ nanocomposite. The overall redox reaction can be represented as

11 Step4:2CTA@ReO4(aq)+CA(aq)+ZnMn2O4(s)→2Re0@ZnMn2O4(s)+oxidized CA(quinone)(aq)+4H2O+2CTA(aq)+

The resulting product was washed through three successive centrifugations at 9000 rpm for 10 min each, using alternating rinses of deionized water and ethanol (99.8%) to eliminate unreacted species. The final solid was dried under vacuum at 60 °C for 12 h, yielding the desired Re@ZnMn_2_O_4_ material, which was stored for electrode fabrication.

### Fabrication of Re@ZnMn_2_O_4_ Modified Electrode for EP Sensing

The surface of the glassy carbon electrode (GCE) was pretreated according to the methods described in the literature.^41,42^ For the fabrication of the Re@ZnMn_2_O_4_-modified GCE used in EP sensing, 15 mg of the Re@ZnMn_2_O_4_ material was dispersed in 5 mL of ultrapure water and subjected to ultrasonic treatment for 25 min to ensure proper homogenization. Subsequently, 6 μL of this suspension was carefully dropped onto the polished surface of the GCE and dried at 55 °C for 20 min. Electrochemical characteristics of the Re@ZnMn_2_O_4_ were investigated using CV and EIS techniques. The various concentration of EP was detected by CV and DPV techniques. The synthesis of ZnMn_2_O_4_ and Re@ZnMn_2_O_4_ and the construction of the Re@ZnMn_2_O_4_ modified GCE to detect EP in human fluids are displayed in Figure 1.

**Figure 1:**
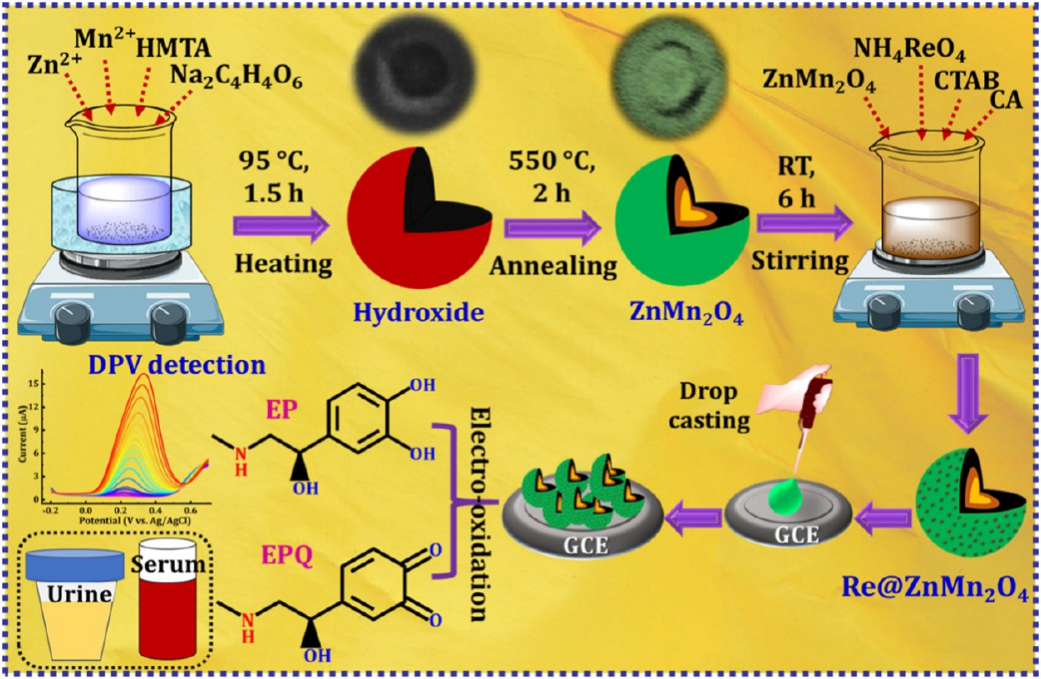
Synthesis of ZnMn_2_O_4_ and Re@ZnMn_2_O_4_ and the construction of the Re@ZnMn_2_O_4_ modified GCE to detect EP in human fluids.

### Preparation of Biological Samples

According to our earlier method,^43^ urine and blood serum samples from healthy donors were centrifuged at 9000 rpm for 20 min to clear debris. Urine samples (1 mL) were diluted to 100 mL and serum samples (2.5 mL) to 25 mL using 0.1 M PBS at pH 7.0, without additional processing. To test real-sample applicability, varying concentrations of EP (10–50 μM) were added to the diluted biological fluids.

## Results and Discussion

### Characterization of ZnMn_2_O_4_ and Re@ZnMn_2_O_4_

The structural morphologies of the ZnMn_2_O_4_ and Re@ZnMn_2_O_4_ were characterized via FESEM and TEM techniques. Figure 2a,b presents FESEM images of ZnMn_2_O_4_ double-shell hollow microspheres at different magnifications. Initially, solid hollow microspheres of ZnMn-hydroxides (Figure S1a) were synthesized via a coprecipitation method, using Na_2_C_4_H_4_O_6_·2H_2_O as a chelating agent and C_6_H_12_N_4_ to gradually release OH^–^ ions. The average particle size of the ZnMn-hydroxide precursor was determined to be 0.97 ± 0.05 μm (Figure S1b) using ImageJ software. Subsequent thermal treatment at 550 °C converted the ZnMn-hydroxides into well-defined ZnMn_2_O_4_ double-shelled hollow microspheres. A low-magnification FESEM image (Figure S2a) shows the ZnMn_2_O_4_ material with an increased average size of 2.25 ± 0.25 μm (Figure S2b). This growth in particle size after calcination is attributed to thermally induced phase transformation, particle sintering, and grain growth during the formation of the crystalline spinel ZnMn_2_O_4_ structure.

**Figure 2:**
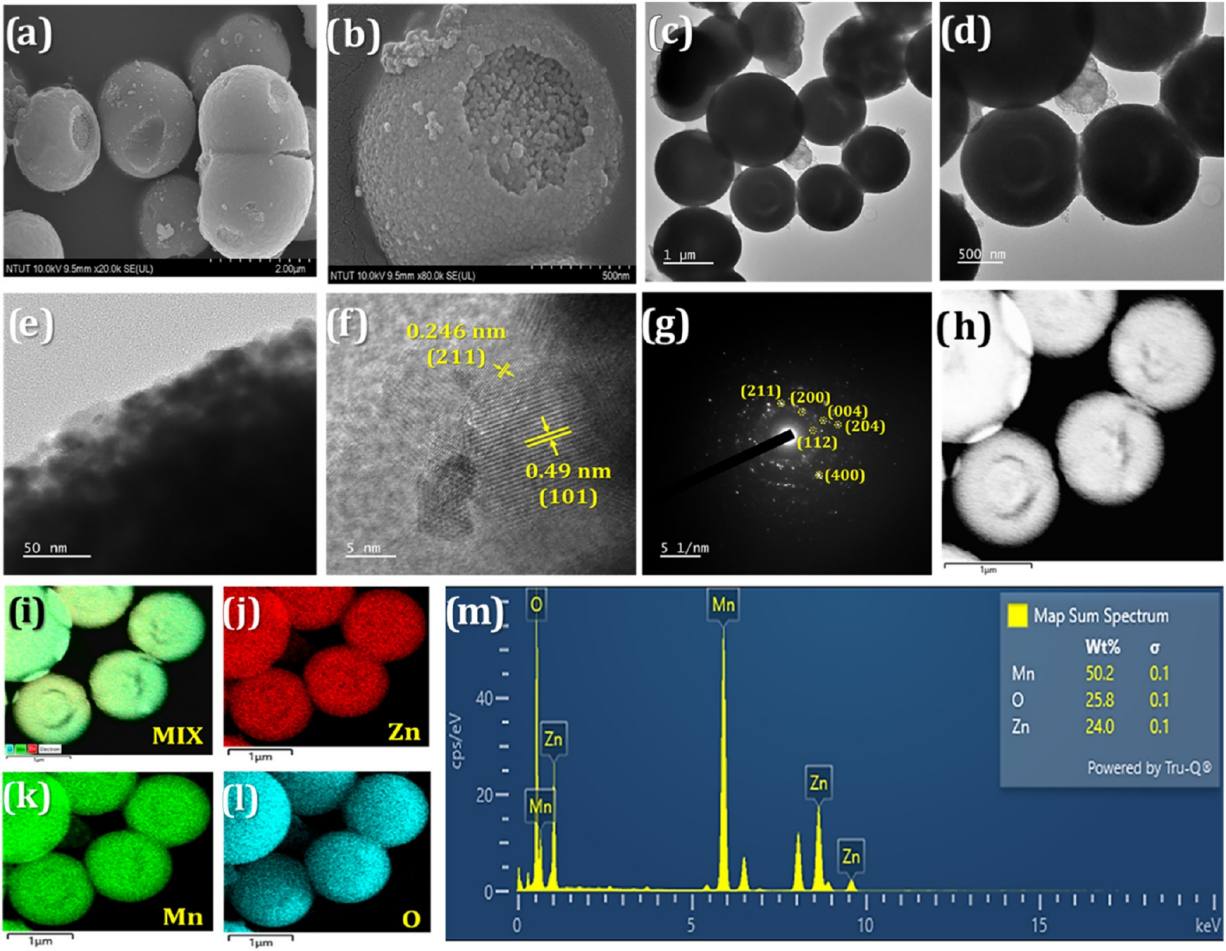
(a–b) FESEM images and (c–e) TEM images of ZnMn_2_O_4_ at diverse magnifications. (f) HRTEM, (g) SAED pattern, and (h) STEM image of ZnMn_2_O_4_ and the elemental mapping of the (i) ZnMn_2_O_4_-mix, (j) Zn, (k) Mn, and (l) O. (m) EDX results of ZnMn_2_O_4_.

Figure 2c–e displays TEM images with varying magnifications, confirming the double-shelled structure of ZnMn_2_O_4_ hollow microspheres. Figure 2f presents an HRTEM image, where lattice fringes with spacings of 0.246 and 0.49 nm correspond to the (211) and (101) crystal planes of ZnMn_2_O_4_, respectively. Figure 2g displays the SAED pattern of ZnMn_2_O_4_, which aligns well with the XRD results, verifying the successful formation and polycrystalline nature of the ZnMn_2_O_4_ phase. Elemental composition and distribution of ZnMn_2_O_4_ were further examined through STEM and elemental mapping, as shown in Figure 2h–l. The results illustrate a homogeneous dispersion of zinc (Zn), manganese (Mn), and oxygen (O) throughout the structure. Additionally, Figure 2m presents the EDS analysis, revealing that Mn, O, and Zn account for 50.2, 25.8, and 24.0% of the total weight, respectively, supporting the desired ZnMn_2_O_4_ product formation.

Figure 3a,b presents FESEM images of Re@ZnMn_2_O_4_, confirming successful encapsulation of Re nanoparticles within the double-shelled hollow structure, while maintaining the original ZnMn_2_O_4_ morphology. Figure 3c–e shows TEM images of Re@ZnMn_2_O_4_, revealing its double-shelled spherical structure, confirming uniform Re nanoparticle distribution on the ZnMn_2_O_4_ surface. HRTEM data (Figure 3f) indicate lattice spacings of 0.271 nm for the (103) plane of ZnMn_2_O_4_ and 0.240 nm for the (100) plane of Re. The SAED pattern in Figure 3g aligns with XRD results. Figure 3h–l shows elemental mapping of Re@ZnMn_2_O_4_-mix, Zn, Mn, O, and Re, while Figure 3m presents EDS data, with weight percentages of 44.6% Mn, 24.1% Zn, 22.0% O, and 9.2% Re, verifying the composition of the Re@ZnMn_2_O_4_ material.

**Figure 3:**
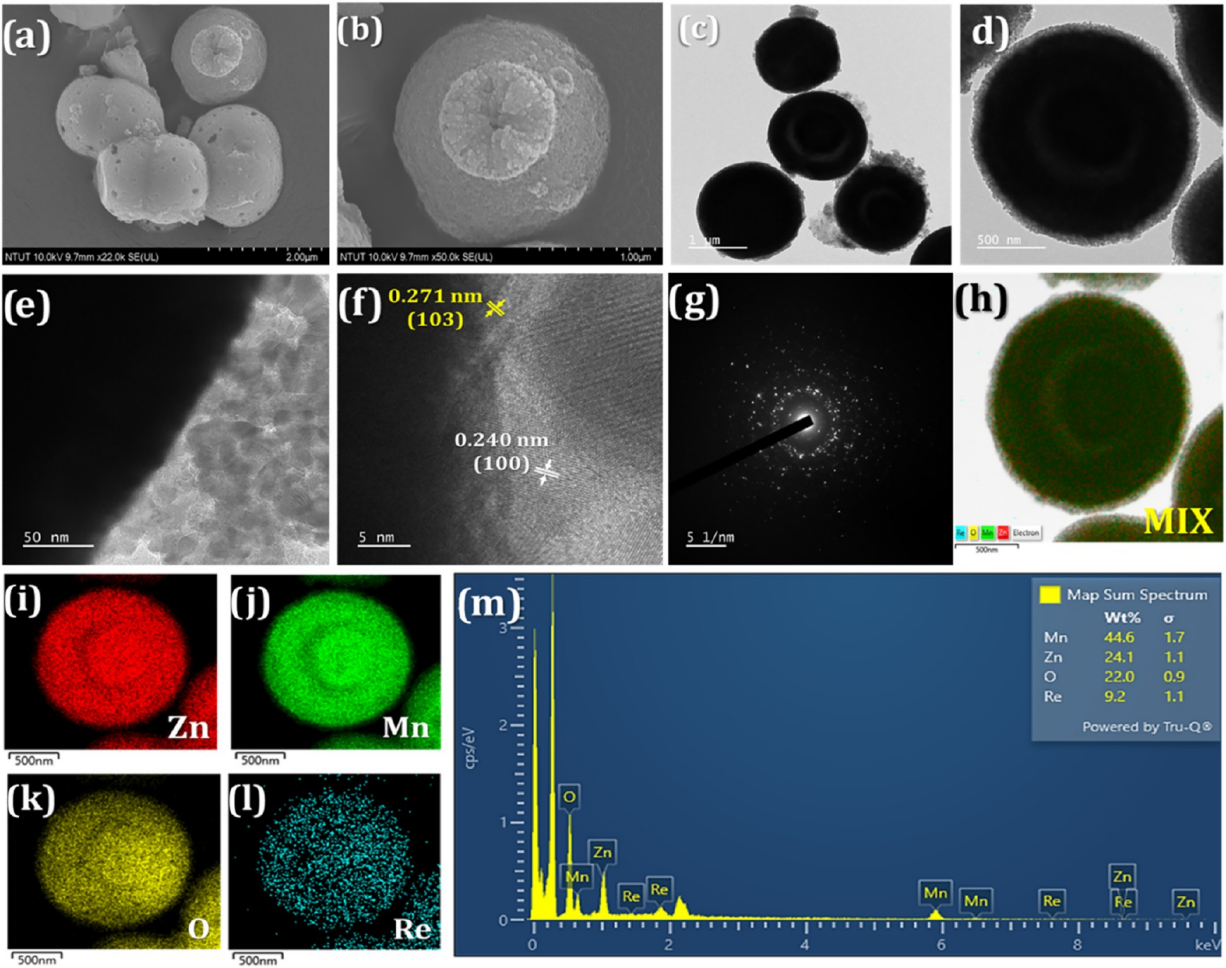
(a–b) FESEM images and (c–e) TEM images of Re@ZnMn_2_O_4_ at diverse magnifications. (f) HRTEM and (g) SAED pattern of Re@ZnMn_2_O_4_, and the elemental mapping of the (h) Re@ZnMn_2_O_4_-mix, (i) Zn, (j) Mn, (k) O, and (l) Re. (m) EDX results of Re@ZnMn_2_O_4_.

Raman spectrum of ZnMn_2_O_4_ (Figure S3) displays three well-defined peaks at 639, 365, and 312 cm^–1^, which are indicative of its spinel structure.^44^ The dominant peak at 639 cm^–1^ is attributed to the symmetric stretching vibration of the tetrahedrally coordinated AO_4_ units, corresponding to the A_1g_ mode. The additional peaks at 365 and 312 cm^–1^ arise from the vibrational modes of oxygen atoms within the octahedral BO_6_ framework.^40^ These features confirm the crystalline ZnMn_2_O_4_ spinel phase formation. Additionally, Figure S4 exhibited the FT-IR spectra of ZnMn_2_O_4_ and Re@ZnMn_2_O_4_. The absorption band at 543 cm^–1^ is attributed to M-O-M stretching vibrations involving both tetrahedral and octahedral metal–oxygen bonds, while the peak at 612 cm^–1^ corresponds to Mn^4+^–O^2–^ bond vibrations in tetrahedral sites, both characteristic of the spinel ZnMn_2_O_4_ structure. A distinct band at 979 cm^–1^ is associated with the C–O stretching vibrations of residual carbonyl groups within the compound. Furthermore, the peaks observed at 1105 and 1204 cm^–1^ are assigned to C–O–C asymmetric stretching and O–H bending vibrations, respectively, indicating the presence of trace organic residues or surface-adsorbed species.^45,46^ All characteristic bands of ZnMn_2_O_4_ were retained in the Re@ZnMn_2_O_4_ spectrum, along with new peaks at 2855 and 2930 cm^–1^, assigned to −CH_2_– and –N^+^(CH_3_)_3_ vibrations from CTAB. Additional signals at 1478, 1581, and 1363 cm^–1^ correspond to Re^0^-related species and C–N stretching modes,^39,47^ confirming the presence of metallic Re nanoparticles in the composite.

Figure 4a illustrates the XRD patterns of pristine ZnMn_2_O_4_ and Re@ZnMn_2_O_4_. The diffraction peaks appearing at 2θ values of 18.05, 29.28, 31.11, 33.03, 36.50, 37.07, 38.99, 44.75, 50.81, 52.05, 54.94, 56.76, 59.16, 60.90, 65.31, and 75.59° are assigned to the (101), (112), (200), (103), (211), (202), (004), (220), (204), (105), (312), (303), (321), (224), (400), and (413) planes, respectively. These reflections are in strong agreement with the standard data from JCPDS card No. 01-071-2499,^40,48^ confirming the tetragonal Hetaerolite structure of ZnMn_2_O_4_, which crystallizes in the *I41/amd* space group (No. 141). The diffraction pattern of Re@ZnMn_2_O_4_ exhibits a nearly identical profile to that of unmodified ZnMn_2_O_4_, indicating that the inclusion of Re does not disrupt the original crystal framework. A minor peak at 2θ = 26.8°, corresponding to the (100) plane of metallic Re (Re^0^), is also observed.^49^ This suggests that Re is integrated into the lattice or surface without forming a separate phase, confirming its successful incorporation while preserving the ZnMn_2_O_4_ structure.

**Figure 4:**
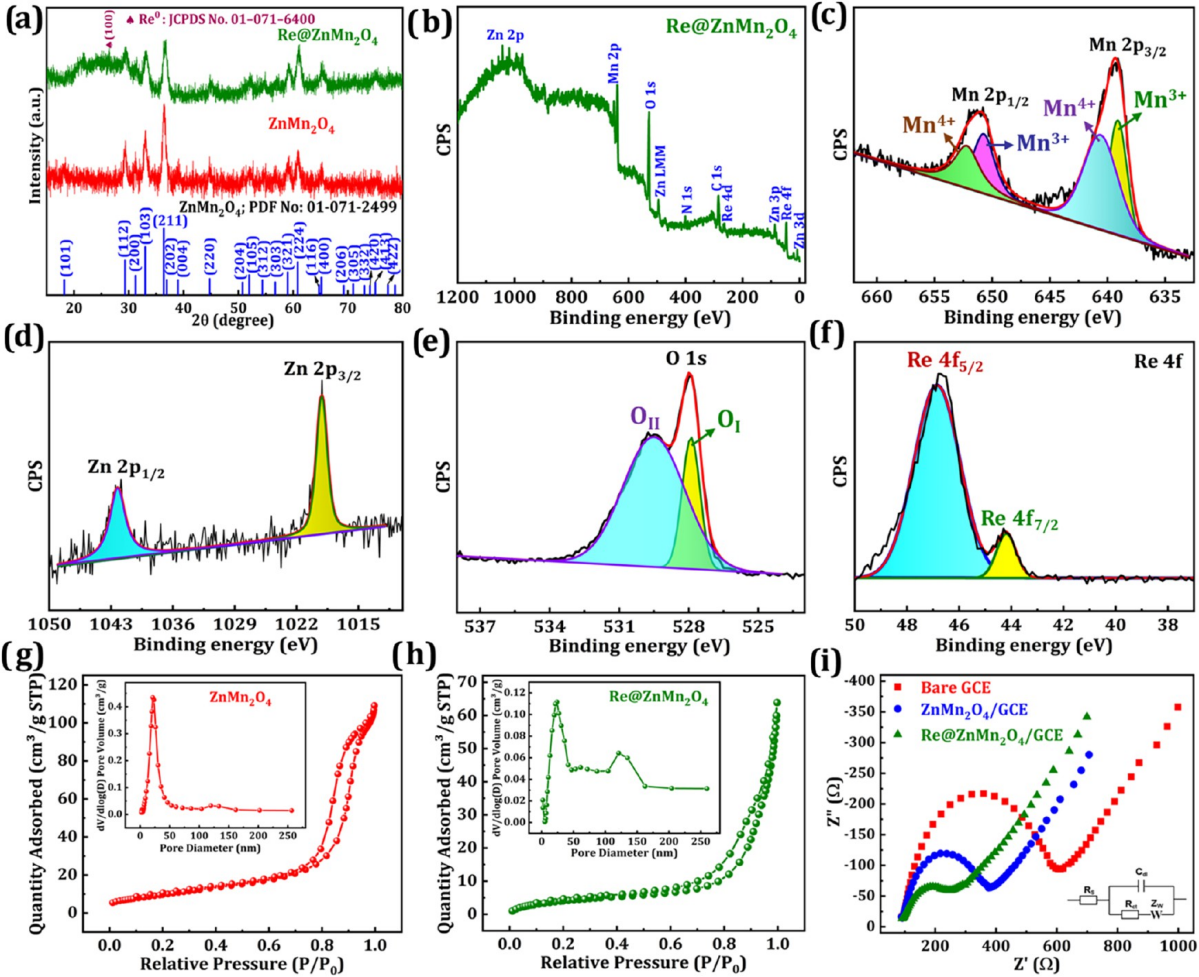
(a) XRD pattern of ZnMn_2_O_4_ and Re@ZnMn_2_O_4_. (b) XPS survey spectrum of Re@ZnMn_2_O_4_ and core-level spectrum of (c) Mn 2p, (d) Zn 2p, (e) O 1s, and (f) Re 4f. BET analysis of (g) ZnMn_2_O_4_ and (h) Re@ZnMn_2_O_4_; inset shows BJH plots. (i) EIS analysis of bare GCE, ZnMn_2_O_4,_ and Re@ZnMn_2_O_4_ modified GCEs.

XPS analysis was conducted to investigate the surface composition and valence states in Re@ZnMn_2_O_4_. As shown in Figure 4b, the survey spectrum confirms the presence of Zn, Mn, O, and Re. In Figure 4c, Mn 2p_3/2_ and 2p_1/2_ peaks at 639.14/650.81 eV and 640.72/652.28 eV correspond to Mn^3+^ and Mn^4+^, indicating a mixed-valence state.^50^ Figure 4d shows Zn 2p_3/2_ and 2p_1/2_ peaks at 1019.15 and 1042.17 eV, consistent with Zn^2+^.^43^ In Figure 4e, the O 1s peak at 527.93 eV (O_I_) is attributed to lattice oxygen, while the 529.58 eV (O_II_) peak suggests adsorbed oxygen species.^51^ Figure 4f shows Re 4f_7/2_ and 4f_5/2_ peaks at 44.09 and 46.76 eV, confirming the presence of metallic Re^0^.^49^ The XPS results verify that Re^0^ was effectively incorporated into the ZnMn_2_O_4_ structure.

The textural properties of ZnMn_2_O_4_ and Re@ZnMn_2_O_4_ were investigated through nitrogen adsorption–desorption isotherms, as illustrated in Figure 4g,h. Both samples displayed type IV isotherms with H2 and H3-type hysteresis loops, indicative of mesoporous characteristics. The BET surface area of pristine ZnMn_2_O_4_ was found to be 36.63 m^2^/g, whereas the Re@ZnMn_2_O_4_ exhibited a reduced surface area of 16.22 m^2^/g. BJH pore size analysis (insets of Figure 4g,h) revealed average pore diameters of about 21.04 nm for ZnMn_2_O_4_ and 23.71 nm for Re@ZnMn_2_O_4_. The observed reduction in surface area following Re modification suggests partial pore blockage or encapsulation of Re species within the mesoporous framework.^52^ Previous reports highlight that mesopores play a crucial role in improving electrochemical behavior by offering abundant active sites.^43^

### Electrochemical Characteristics of ZnMn_2_O_4_ and Re@ZnMn_2_O_4_

Electrochemical impedance spectroscopy (EIS) was utilized to examine the interfacial interactions and electron transfer behavior between the electrode surface and solution, using the [Fe­(CN)_6_]^3–/4–^ redox couple. Figure 4i displays the Nyquist plots for the bare GCE, ZnMn_2_O_4_/GCE, and Re@ZnMn_2_O_4_/GCE. In these spectra, the semicircular region corresponds to the charge transfer resistance (*R*_ct_). At the same time, the low-frequency linear portion specifies diffusion control, with *R*_ct_ reflecting the efficiency of electron transfer at the electrode–electrolyte interface. The measured *R*_ct_ values were 513 Ω for the bare GCE, 287 Ω for ZnMn_2_O_4_/GCE, and 175 Ω for Re@ZnMn_2_O_4_/GCE, as shown in Figure S5. The significantly lower *R*_ct_ observed for Re@ZnMn_2_O_4_/GCE indicates improved interfacial conductivity and enhanced electron transport, confirming the effective integration of Re into the ZnMn_2_O_4_ matrix.

Cyclic voltammetry (CV) analysis was initially performed to evaluate the electrochemical performance of both unmodified and modified electrodes using a [Fe­(CN)_6_]^3–/4–^ redox solution at a scan sweep (υ) of 0.05 V/s. Figure 5a illustrates the electrocatalytic behavior of the blank GCE, ZnMn_2_O_4_/GCE, and Re@ZnMn_2_O_4_/GCE. The bare GCE exhibits a pair of redox peaks with a peak-to-peak separation (Δ*E*_p_) of 136 mV and anodic and cathodic peak currents (*I*_pa_ and *I*_pc_) of 70 μA and −67 μA, respectively, resulting in a reversible redox system. In contrast, ZnMn_2_O_4_/GCE shows an improved electrochemical response, with a lesser Δ_Ep_ of 84 mV and inclined peak currents (*I*_pa_ = 75 μA, *I*_pc_ = −81 μA), suggesting enhanced electron transfer relative to the bare electrode. Notably, Re@ZnMn_2_O_4_/GCE exhibits the best performance with a Δ_Ep_ of 82 mV and even higher peak currents (*I*_pa_ = 85 μA, *I*_pc_ = −87 μA), indicating superior electrocatalytic activity and more efficient electron transfer between the Re@ZnMn_2_O_4_/GCE and [Fe­(CN)_6_]^3–/4–^ probe.

**Figure 5:**
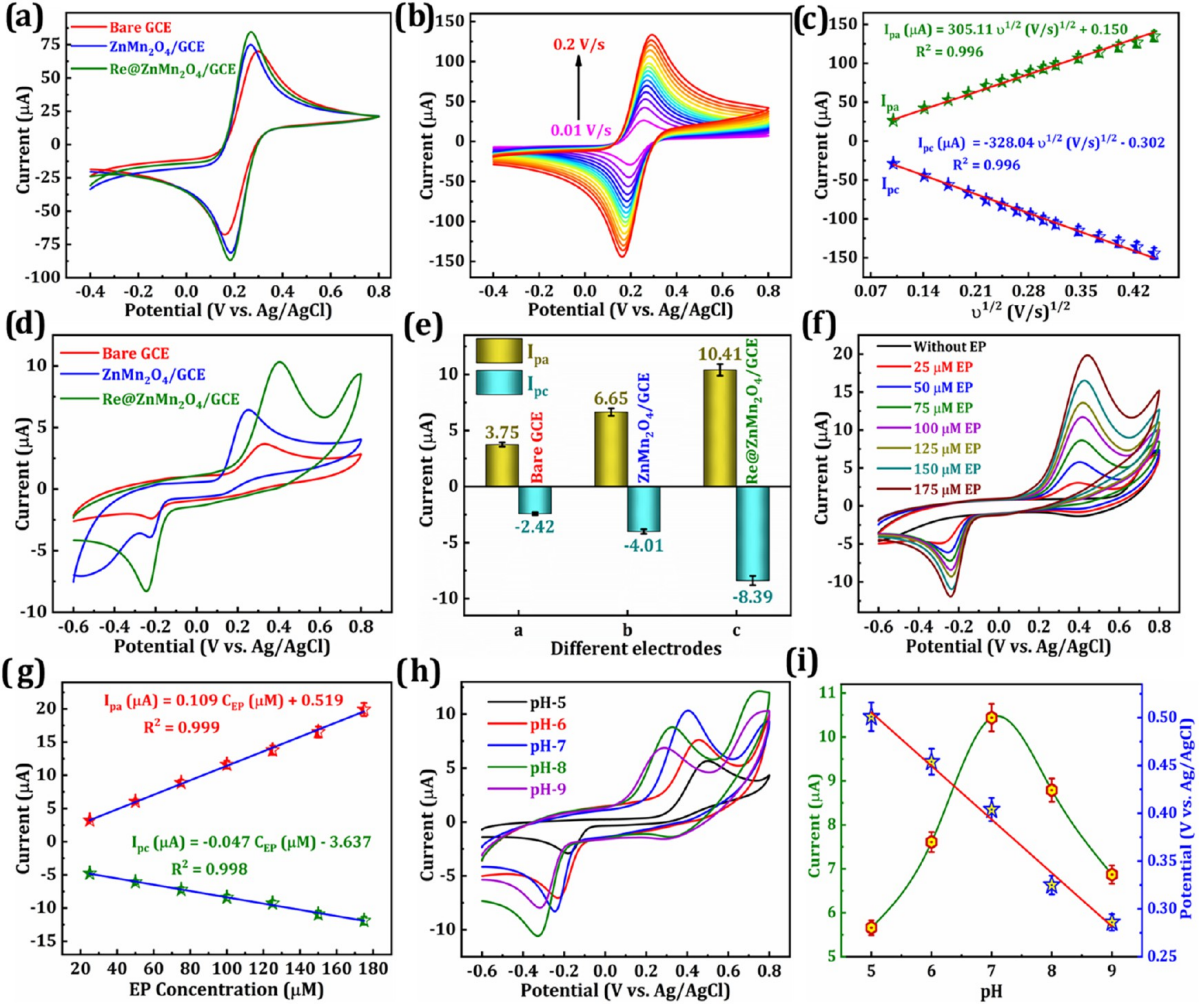
(a) CV curves of bare GCE, ZnMn_2_O_4_/GCE, and Re@ZnMn_2_O_4_/GCE in a 5 mM [Fe­(CN)_6_]^3–/4–^ + 100 mM KCl. (b) CV curves of increasing scan sweep (υ: 0.01–0.2 V/s) at Re@ZnMn_2_O_4_/GCE in 5 mM [Fe­(CN)_6_]^3–/4–^ + 100 mM KCl and the (c) linear plot of υ^1/2^ against *I*_pc_ and *I*_pa_. (d) CV responses of bare GCE, ZnMn_2_O_4_/GCE, and Re@ZnMn_2_O_4_/GCE for 100 μM EP detection with (e) corresponding bar chart. (f) CVs of without EP and increasing concentrations of EP at Re@ZnMn_2_O_4_/GCE, and (g) linear plot of EP concentration against current. (h) CV curves of the diverse pH from 5 to 9 with their current response, and (i) the plot of pH vs E_pa_ and pH vs *I*_pa_.

Figure 5b presents the CV curves of Re@ZnMn_2_O_4_/GCE at varying scan sweeps (υ = 0.01–0.2 V/s). As the υ increases, both *I*_pa_ and *I*_pc_ are inclined proportionally. Figure 5c shows the linear relationship between *I*_pa_ and *I*_pc_ versus the υ^1/2^ (V/s)^1/2^, confirming a diffusion-controlled redox process at the Re@ZnMn_2_O_4_/GCE–electrolyte interface. The regression equations are as follows:

12 $$I_{\mathrm{pa}}\,\;\left(\mu A\right)=305.11\;\upsilon^{1/2}{\left(V/s\right)}^{1/2}+0.150\;\left(R^{2}=0.996\right)$$

13 $$I_{\mathrm{pc}}\;\left(\mu A\right)=-328.04\;\upsilon^{1/2}{\left(V/s\right)}^{1/2}-0.302\;\left(R^{2}=0.996\right)$$

The Randles-Sevcik formula was utilized to find the electroactive surface area (EASA) of bare GCE, ZnMn_2_O_4_/GCE, and Re@ZnMn_2_O_4_/GCE, which were measured in a redox solution.^53^

14 $$I_{p}=2.69\times 10^{5}\;A\;C\;n^{3/2}D^{1/2}\upsilon^{1/2}$$

Here, the EASA (cm^2^), the electron transfer *n* is (usually 1), the analyte diffusion coefficient is *D* (7.26 × 10^–6^ cm^2^ s^–1^), υ (0.05 V/s), and the electrolyte concentration is *C* (5 × 10^–6^ mol cm^–3^). According to the calculation, the EASA is 0.083 cm^2^ for bare GCE, 0.090 cm^2^ for ZnMn_2_O_4_/GCE, and 0.102 cm^2^ for Re@ZnMn_2_O_4_/GCE, suggesting Re@ZnMn_2_O_4_/GCE has a larger active surface area with greater electric conductivity characteristics.

### Electrochemical Response of Modified Electrodes with EP Concentrations

The CV curves of bare, ZnMn_2_O_4_, and Re@ZnMn_2_O_4_ modified GCEs were measured in a 100 μM EP solution containing 0.1 M PBS at pH 7.0, as displayed in Figure 5d. The current response is illustrated in the bar chart of Figure 5e. The unmodified GCE recorded an *I*_pa_ of 3.75 μA and *I*_pc_ of −2.42 μA, demonstrating quasi-reversible EP. When modified with ZnMn_2_O_4_, the ZnMn_2_O_4_/GCE demonstrated increased redox activity, with *I*_pa_ and *I*_pc_ values of 6.65 μA and −4.01 μA, respectively, than bare GCE. The Re@ZnMn_2_O_4_/GCE exhibited the most pronounced electrochemical response, achieving an *I*_pa_ of 10.41 μA and an *I*_pc_ of −8.39 μA. These results highlight the superior redox behavior and enhanced electron transfer capability of the Re-doped ZnMn_2_O_4_ hybrid.

The electroanalytical characteristics and redox response of Re@ZnMn_2_O_4_/GCE were further examined using CV, as presented in Figure 5f. Both the absence of EP and the presence of increasing EP concentrations, ranging from 25 to 175 μM, were tested in a 0.1 M PBS at pH 7.0. As the concentration of EP increased, a corresponding rise in redox peak current was observed, confirming a concentration-dependent electrochemical response. This behavior indicates that the Re@ZnMn_2_O_4_/GCE exhibits high sensitivity and effective electrocatalytic activity for the oxidation and reduction of EP. The variation in peak currents directly reflects the redox behavior of EP at diverse concentration levels. Figure 5g presents a linear plot of EP concentration against the *I*_pa_ and *I*_pc_, along with the regression equations.

$$I_{\mathrm{pa}}\left(\mu A\right)=0.109\;C_{\mathrm{EP}}\;\left(\mu M\right)+0.519;\;R^{2}=0.999$$ 15

$$I_{\mathrm{pc}}\left(\mu A\right)=-0.047\;C_{\mathrm{EP}}\;\left(\mu M\right)-3.637;\;R^{2}=0.998$$ 16

The enhanced EP sensing capability of Re@ZnMn_2_O_4_ provides excellent electrical conductivity, numerous reactive sites, a greater surface area, and high electrocatalytic activity.

### Influence of pH on EP

To investigate the influence of pH on the sensing performance of the Re@ZnMn_2_O_4_/GCE toward EP, CV was performed in PBS across a pH range of 5 to 9. As illustrated in Figure 5h, the most pronounced redox response was observed at pH 7. The optimum pH observed for EP detection was higher than the commonly reported range of pH 7–8, and notably above the pH 5 previously used with cobalt phthalocyanine/single-walled carbon nanotube-based electrodes.^54^ In aqueous media (pH > 3.0), its oxidation follows an ECE mechanism, with each step transferring two protons and two electrons reversibly.^55,56^ EP contains hydroxyl groups with p*K*_a_ values ranging from 8.7 to 12 and an amino group with a p*K*_a_ of approximately 9.9,^54^ which indicates partial ionization in aqueous media and a weakly acidic character. At higher pH values, the hydroxyl groups of EP tend to deprotonate, forming negatively charged –O^–^ species. These anionic forms are electrostatically attracted to positively charged regions on the Re@ZnMn_2_O_4_/GCE surface, which contains Zn^2+^ and redox-active Re^5+^/^7+^ centers. At physiological pH (∼7), this electrostatic interaction enhances EP adsorption and detection. Thus, pH 7 was selected for subsequent analysis due to both biological relevance and optimal sensor response.^57^

Figure 5i further demonstrates the variation in oxidation current (*I*_pa_) and potential (*E*_pa_) as a function of pH, while the *E*_pa_ shows a linear shift with pH, following the equation:

17 $$E_{\mathrm{pa}}\left(V\right)=-0.056\;\mathrm{pH}+0.785\left(R^{2}=0.988\right)$$

The observed slope of 56 mV/pH is close to the theoretical Nernstian value of 59 mV/pH, signifying that the transfer of protons and electrons proceeds in equal numbers. Since EP undergoes a two-electron oxidation, it is inferred that two protons participate in the reaction as well.^58^

### Investigation of Scan Rate Influence

The electrocatalytic behavior and kinetics of the Re@ZnMn_2_O_4_/GCE was assessed by varying the scan speed from 0.02 to 0.2 V/s in a 100 μM EP solution (Figure 6a). Both *I*_pa_ and *I*_pc_ rose with increasing υ, accompanied by minor shifts in peak potentials. Lower scan speeds allow a thicker diffusion layer due to extended diffusion time, while higher rates restrict diffusion, enhancing analyte transport and current.^59^ As depicted in Figure 6b, peak current varies linearly with the SQRT of the υ, confirming diffusion-controlled kinetics.^60^ The regression equations are

$$I_{\mathrm{pa}}(\mu A)=57.824\;\upsilon^{1/2}\;\left(V/s\right)-2.946;\;R^{2}=0.997$$ 18

$$I_{\mathrm{pc}}\left(\mu A\right)=-33.604\;\upsilon^{1/2}\;\left(V/s\right)-0.846;\;R^{2}=0.999$$ 19

**Figure 6:**
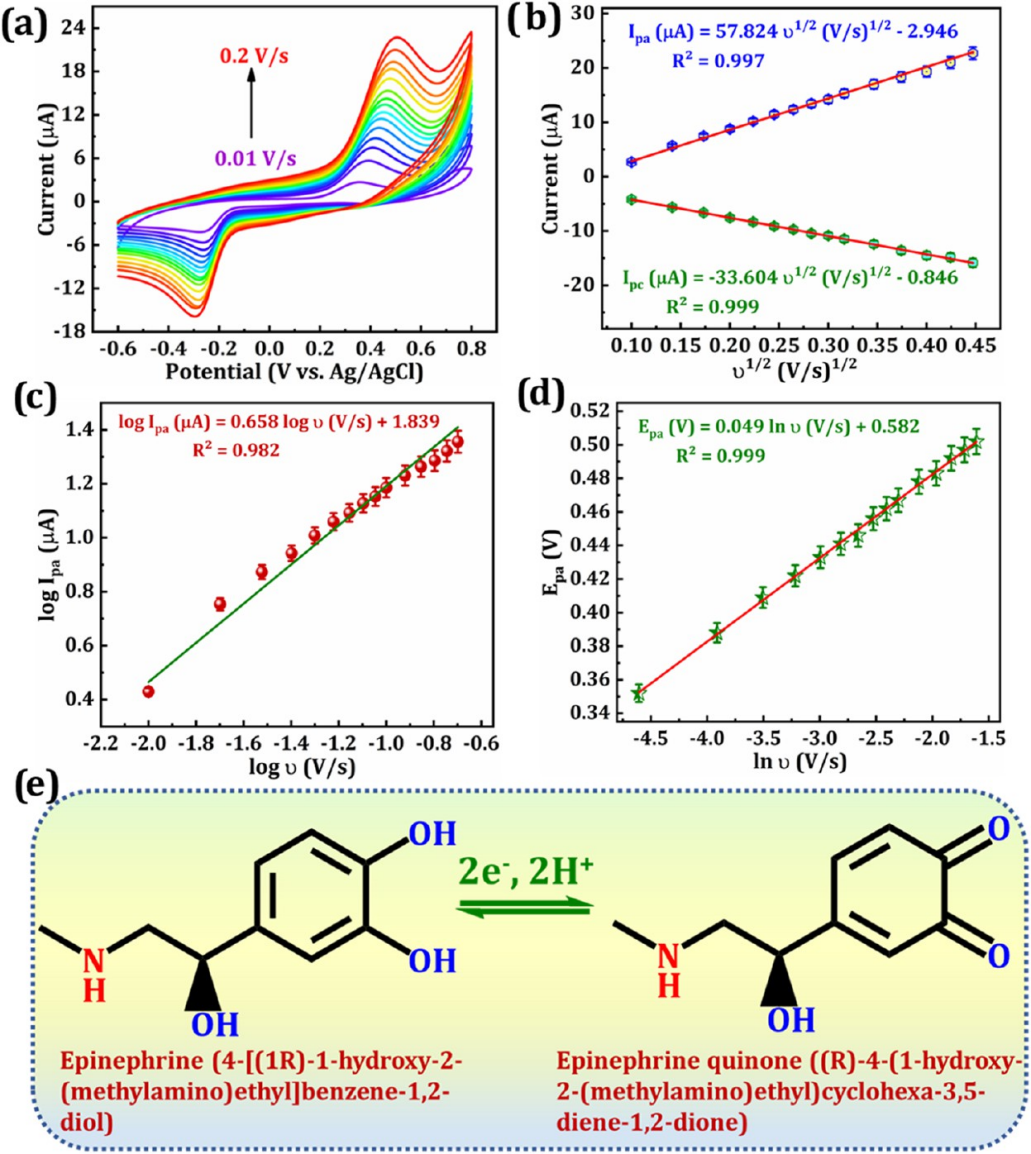
(a) CVs obtained with Re@ZnMn_2_O_4_/GCE for diverse υ from 0.01 to 0.2 V/s in PBS (pH = 7) containing 100 μM EP and (b) linear plot of the SQRT υ versus the current. Linear plots of the (c) log υ versus the log *I*_pa_ and (d) ln υ versus *E*_pa_. (e) The Electrochemical reaction mechanism of EP.

The Re@ZnMn_2_O_4_/GCE demonstrates significant affinity for EP molecules, likely due to bonding with the hydroxyl groups present in EP. As shown in Figure 6c, there is a distinct linear correlation between the log υ and the log *I*_pa_, expressed by the following equations:

20 $${\mathrm{Log}I}_{\mathrm{pa}}\left(\mu A\right)=0.658\;\log\upsilon \;\left(V/s\right)+1.839;R^{2}=0.982$$

The slope of 0.658 is close to the theoretical value of 0.5,^61^ supporting that the oxidation of EP is mainly governed by a diffusion-controlled mechanism.^62^ Moreover, the charge transfer coefficient (α) and electron transfer rate constant (*k*_s_) were derived from the Laviron equation. According to the Laviron model, such behavior can be described by the linear equation obtained from a plot of the natural logarithm of υ (ln υ) and the peak potential (*E*_pa_) (Figure 6d).

21 $$E_{\mathrm{pa}}\left(V\right)=0.049\;\ln\upsilon \;\left(V/s\right)+0.582;R^{2}=0.999$$

The obtained slope and intercept values are substituted into Laviron’s model.^63^

22 $${\log k}_{s}=\alpha \;\log\left(1-\alpha \right)+\left(1-\alpha \right)\log\alpha -\log\frac{\mathrm{RT}}{n\;F\upsilon }-\frac{\alpha \left(1-\alpha \right)\mathrm{nF}\Delta E_{p}}{2.303\;\mathrm{RT}}$$

Here, *E*_p_ is the anodic peak potential, *E* ^0^ represent the formal potential, *R* indicates the gas constant, *T* is the room temperature, α represent the electron transfer coefficient, n denotes the charge transfer number, *F* is the faraday’s constant, *k*_s_ represents the electron transfer rate constant of the surface process, υ indicates the scan rate, and the *E* ^0^ could be known as intercept of the calibration plot. Using eq 22, the α and *k*_s_ values were determined to be 0.54 and 3.6 × 10^–3^ s^–1^, indicating that EP and EPQ are interconverted through a two-electron (2e^–^) transfer process.^64^

### Electrooxidation Mechanism of EP

The oxidation mechanism of EP in PBS using the Re@ZnMn_2_O_4_/GCE is illustrated in Figure 6e. The CV data demonstrate a quasi-reversible redox reaction, with a stronger oxidative response compared to the reductive one (Figure 5d,f,h), aligning with established findings. As indicated in Figure 6a, the process is diffusion-controlled and involves an equal number (1:1) transfer of electrons and protons. During the forward scan from – 0.6 to 0.8 V, EP is oxidized, transforming its hydroxyl groups into a quinone form (EPQ). On the reverse scan, EPQ is reduced back to EP at the electrode surface through a 2e^–^, 2H^+^ transfer pathway.^65,66^

### Electroanalytical Quantification of EP via DPV

The superior sensing ability of the Re@ZnMn_2_O_4_/GCE was validated through the quantitative analysis of EP in PBS (pH 7) using DPV, conducted under optimized electrode and experimental conditions. To generate the calibration curve, standard EP solutions were incrementally added into the PBS electrolyte, and the resulting DPV signals were measured. As illustrated in Figure 7a, well-defined anodic peaks emerged within the potential range of −0.2 to 0.7 V, with increasing peak intensities corresponding to EP concentrations ranging from 0.5 to 3751 μM, confirming a clear dependence on concentration. Figure 7b presents the corresponding calibration graph (0.5–1950.3 μM), revealing two distinct linear segments: *I*_pa1_ at lower concentrations (0.5–275.3 μM) and *I*_pa2_ at higher concentrations (275.3–1950.3 μM). At low EP concentrations, molecular movement is rapid, leading to a swift electrochemical response. In contrast, higher EP concentrations significantly hinder molecular diffusion, resulting in two distinct linear regions in the calibration curve,^67^ each is described by its respective regression equations:

23 $$I_{\mathrm{pa}1}\;(\mu A)=0.02{\;C}_{\mathrm{EP}}\;\left(\mu M\right)+0.635\;\left(R^{2}=0.997\right)$$

24 $$I_{\mathrm{pa}2}\;\left(\mu A\right)=0.004\;C_{\mathrm{EP}}\;\left(\mu M\right)+4.965\;\left(R^{2}=0.991\right)$$

**Figure 7:**
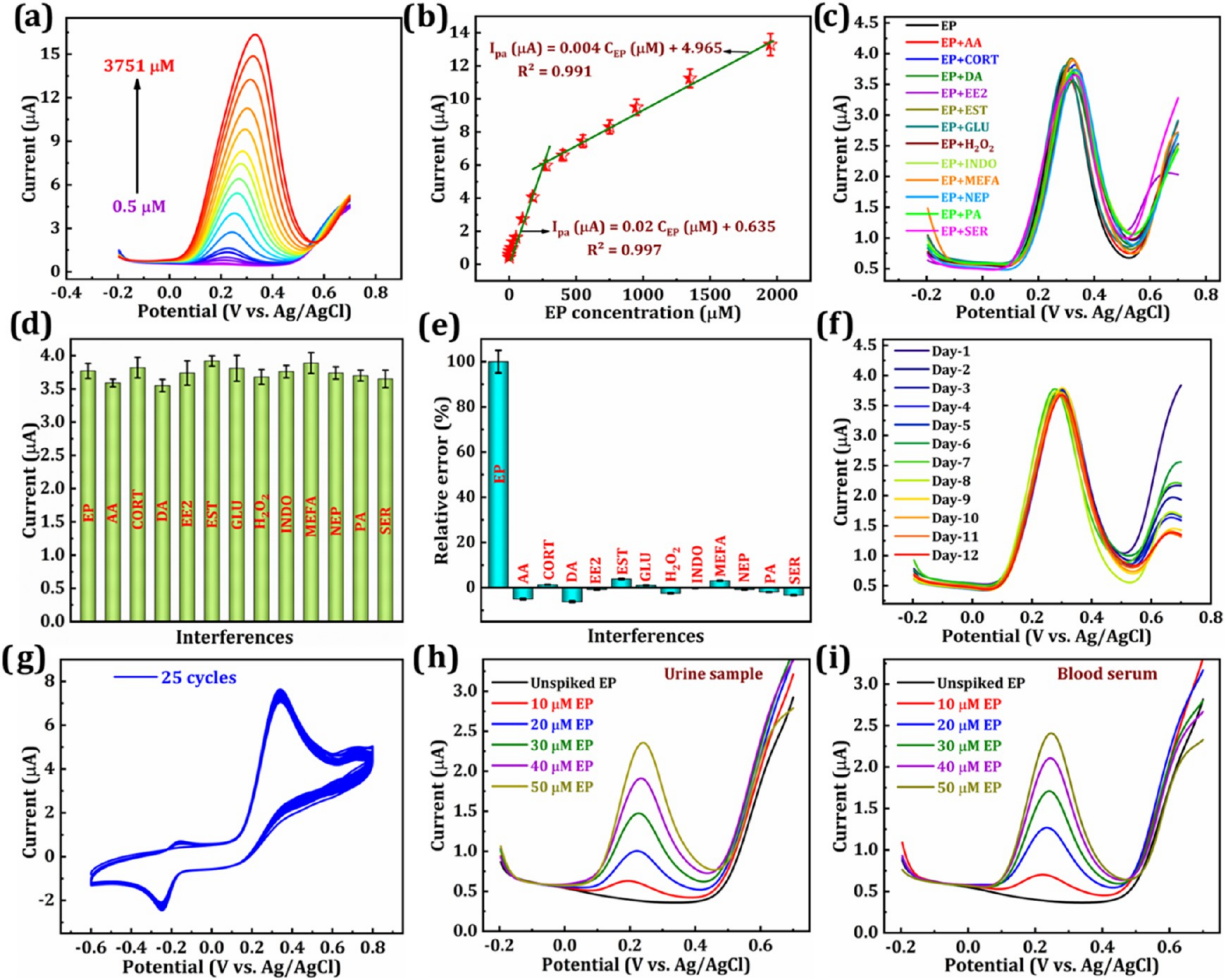
(a) DPV response at the Re@ZnMn_2_O_4_/GCE on addition of increasing concentrations of EP (0.5–3751 μM). (b) Linear plot of EP concentration (0.5–1950.3 μM) vs *I*_pa_. (c) DPV curves of the EP response with interferences and the bar chart of (d) current values and (e) relative error (%). (f) storage stability, and (g) cyclic stability of the EP sensor. Real-time analysis of (h) urine and (i) blood serum samples.

The LOD was determined to be 0.21 μM (LOD = 3S/b),^20^ with the calculated sensitivity of 0.282 μA μM^–1^ cm^–2^ for EP sensing, revealing reliable EP sensing performance throughout a wide concentration interval. Table 1 highlights the analytical performance of existing electrochemical methods for EP detection, showing that the proposed sensor outperforms previously reported constructed electrodes in terms of sensitivity and detection range. Furthermore, its uncomplicated fabrication process contributes to its potential for practical implementation.

**Table 1:** Electroanalytical Characteristics of the Re@ZnMn_2_O_4_/GCE Compared with Previous Reports for EP Detection

**electrode material**	**electrochemical technique**	**linear range (μM)**	**LOD (μM)**	**refs**
PDA@CeO_2_/GCE	DPV	20–100	8.37	^5^
MWCNT/Fe_3_O_4_/Nc/GCE	DPV	7.5–48	12.3	^56^
CQDs/CuO/GCE	SWV	10–100	15.99	^68^
MWCNTs/poly-FA/GCE	AP	73–1406	22.2	^69^
Gold Nanoporous Film	CV	50–1000	19	^66^
GCE-CNF-AuNPs	SWV	50–1000	1.7	^58^
Poly(EBT)-GCE	DPV	2.5–50	0.3	^70^
Poly(caffeic acid)-GCE	CV	2–80	0.2	^71^
Bare IPGE	DPV	0.8–10	0.34	^72^
β-CD/NRGO-ZnFe_2_O_4_/Pt	SWV	4–100	1.12	^62^
rGO-NiO/ITO	SWV	0.5– 50.0	0.423	^73^
NiO-rGO/GCE	CV	50–1000	10	^74^
**Re@ZnMn**_**2**_ **O**_**4**_ **/GCE**	**DPV**	**0.5–1950.3**	**0.21**	**this work**

Notably, in comparison with previously reported metal oxide-based sensors for EP detection,^5,56,62,68,73,74^ the Re@ZnMn_2_O_4_/GCE achieves a wider linear detection range and a significantly lower LOD. This enhanced performance can be attributed to the material’s excellent electrocatalytic properties, including abundant active sites, strong conductivity, and superior catalytic efficiency. In addition, the Re@ZnMn_2_O_4_/GCE displays consistent response stability, along with excellent precision, making it a promising platform for sensitive and reliable EP analysis.

### Selectivity Assessment

To evaluate the ability of the Re@ZnMn_2_O_4_/GCE to selectively and specifically detect EP (100 μM), interference experiments were conducted using DPV. A range of potential interfering substances, including small molecules and drugs commonly found in environmental and biological contexts, was tested. These substances included ascorbic acid (AA), cortisol (CORT), dopamine (DA), ethinyl estradiol (EE2), estradiol (EST), glucose (Glu), hydrogen peroxide (H_2_O_2_), mefenamic acid (MEFA), norepinephrine (NEP), paracetamol (PA), indomethacin (INDO), and serotonin (SER), with each being added in 4-fold. As revealed in Figure 7c, the current responses for each substance were recorded, and the bar chart in Figure 7d summarizes these results. The data indicate that the sensor signal remained stable despite the presence of these substances. Furthermore, Figure 7e illustrates that the relative error (RE, %) in EP detection under interference conditions consistently remained below 6.5%. These findings demonstrate the sensor’s robust selectivity and specificity for EP, even when tested alongside structurally or chemically similar compounds.

### Stability (Storage and Cyclic), Reproducibility, and Repeatability

To assess the durability of the EP sensor over time, a 12-day storage evaluation was conducted. The Re@ZnMn_2_O_4_/GCE was stored at 4 °C and monitored using DPV measurements, as displayed in Figure 7f. After 12 days, the sensor preserved 98.0% of its initial current output, with stable peak positions and consistent signal patterns. As depicted in Figure S6, the current response showed a slight decrease from 3.78 to 3.69 μA, confirming suitability for extended use. In addition, Figure 7g displays the cyclic stability of the Re@ZnMn_2_O_4_/GCE. Upon completion of 25 continuous CV scans, the sensor showed minimal signal loss, retaining 99.0% of the current, which highlights its excellent stability for EP sensing.

To verify the analytical reliability of the constructed EP sensor, both reproducibility and repeatability assessments were performed. Reproducibility was confirmed by modifying six GCEs with Re@ZnMn_2_O_4_ under identical conditions, yielding consistent CV responses with an RSD of 2.21% (Figure S7a). Repeatability was assessed through eight consecutive CV scans on a Re@ZnMn_2_O_4_/GCE, showing highly overlapping curves with an RSD of 2.8% (Figure S7b). These findings confirm the strong measurement precision and dependable performance of the Re@ZnMn_2_O_4_-based sensor.

### Detection in Actual Samples

The practical utility of the Re@ZnMn_2_O_4_-modified sensor was assessed by analyzing real biological samples using DPV, and the results for blank urine and blood serum samples, along with their tested responses, are shown in Figure 7h,i. First, human urine and serum samples were diluted with 0.1 M PBS, pH 7.0, followed by the addition of EP at varying concentrations (10–50 μM). As illustrated in Figure 7h, the urine sample exhibited a distinct oxidation peak, with the current response increasing proportionally with EP concentration, and a linear relationship is depicted in Figure S8a. A similar trend was observed in the serum sample, as presented in Figure 7i, with its linear relationship displayed in Figure S8b. The sensor exhibited consistent recovery and low variability, as illustrated in Table S1. These findings signify that the Re@ZnMn_2_O_4_-based sensor exhibited robust detection ability even in complex physiological samples.

## Conclusions

This work reports the stepwise synthesis of Re@ZnMn_2_O_4_ hollow microspheres by first preparing self-templated double-shelled ZnMn_2_O_4_ hollow microspheres via a coprecipitation and annealing route, followed by Re NPs loading through a CA-assisted reduction strategy. The resulting Re@ZnMn_2_O_4_ modified GCE exhibits a high electrochemical performance for EP detection. Comprehensive structural and electrochemical characterization confirmed the formation of a well-defined hollow architecture with enhanced charge transfer and redox properties. The oxidation of EP followed a diffusion-controlled mechanism involving a 2e^–^/2H^+^ transfer under physiological pH conditions. The sensor demonstrated a wide linear range (0.5–1950.3 μM), low LOD (0.21 μM), and good sensitivity (0.282 μA μM^–1^ cm^–2^), along with excellent selectivity (RE < 6.5%) and operational stability (98% signal retention over 12 days; 99.0% over 25 cycles). Reproducibility and repeatability were confirmed with low RSD values, and reliable detection was achieved in human urine and serum. This study shows that CA-mediated sustainable nanostructures enable sensitive biosensing and could be integrated into portable or wearable devices for real-time monitoring.

## Supplementary Material



## Notes

Chemicals and reagents; characterization techniques; FESEM image and particle size distribution diagram of ZnMn-hydroxide and ZnMn_2_O_4_; Raman spectrum of ZnMn_2_O_4_; FT-IR spectrum of ZnMn_2_O_4_ and Re@ZnMn_2_O_4_; bar chart comparing *R*_ct_ values for different electrodes; storage stability plot; CV curves for reproducibility and repeatability analyses; linear calibration plots for real sample analysis; Table S1. EP levels in biological samples (PDF)

## References

[1] Rehman S. U., Musuvadhi Babulal S., Wu H.-F. (2024). Oxygen-Deficient Mn _2_ O _3_ Nanosheets for Dual Colorimetric and Electrochemical Detection
of Epinephrine. ACS Appl. Nano Mater..

[2] Tadi K. K., Motghare R. V., Ganesh V. (2015). Electrochemical Detection of Epinephrine
Using a Biomimic Made up of Hemin Modified Molecularly Imprinted Microspheres. RSC Adv..

[3] Lavanya N., Fazio E., Neri F., Bonavita A., Leonardi S. G., Neri G., Sekar C. (2015). Simultaneous
Electrochemical Determination
of Epinephrine and Uric Acid in the Presence of Ascorbic Acid Using
SnO2/Graphene Nanocomposite Modified Glassy Carbon Electrode. Sens. Actuators, B.

[4] Wang Q., Si H., Zhang L., Li L., Wang X., Wang S. (2020). A Fast and
Facile Electrochemical Method for the Simultaneous Detection of Epinephrine,
Uric Acid and Folic Acid Based on ZrO2/ZnO Nanocomposites as Sensing
Material. Anal. Chim. Acta.

[5] Yadav S., Sadique M. A., Singhai S., Khan R. (2023). Rapid Electrochemical
Detection of Epinephrine in Human Serum Based on Polydopamine-Wrapped
Cerium Oxide Nanocomposite. Hybrid Adv..

[6] Sainz R., del Pozo M., Vilas-Varela M., Castro-Esteban J., Pérez Corral M., Vázquez L., Blanco E., Peña D., Martín-Gago J. A., Ellis G. J., Petit-Domínguez M. D., Quintana C., Casero E. (2020). Chemically Synthesized Chevron-like
Graphene Nanoribbons for Electrochemical Sensors Development: Determination
of Epinephrine. Sci. Rep..

[7] Mishra A. (2009). Simultaneous
Determination of Epinephrine and Norepinephrine by High Performance
Liquid Chromatography. Sci. Pharm..

[8] AL-Ayash Ashraf. S., Muhamad Y. H., Ghafouri S. A. (2025). Spectrophotometric
Determination
of Epinephrine in Pharmaceutical Preparations Using Praseodymium as
Mediating Metals. Baghdad Sci. J..

[9] Nevado J. J. B., Gallego J. M. L., Laguna P. B. (1995). Spectrophotometric
Determination of Catecholamines with Metaperiodate by Flow-Injection
Analysis. Anal. Chim. Acta.

[10] Zhao Y., Zhao S., Huang J., Ye F. (2011). Quantum Dot-Enhanced
Chemiluminescence Detection for Simultaneous Determination of Dopamine
and Epinephrine by Capillary Electrophoresis. Talanta.

[11] Guo Y., Yang J., Wu X., Du A. (2005). A Sensitive Fluorimetric
Method for the Determination of Epinephrine. J. Fluoresc..

[12] Kiranmai S., Kuchi C., Sravani B., Luczak T., Kim M. J., Madhavi G., Veera Manohara Reddy Y. (2022). Construction
of Ultrasensitive
Electrochemical Sensor Using TiO2-Reduced Graphene Oxide Nanofibers
Nanocomposite for Epinephrine Detection. Surf.
Interfaces.

[13] Yadav S., Parihar A., Sadique M. A., Ranjan P., Kumar N., Singhal A., Khan R. (2023). Emerging Point-of-Care Optical Biosensing
Technologies for Diagnostics of Microbial Infections. ACS Appl. Opt. Mater..

[14] Li Z., Guo Y., Yue H., Gao X., Huang S., Zhang X., Yu Y., Zhang H., Zhang H. (2021). Electrochemical Determination of
Epinephrine Based on Ti3C2Tx MXene-Reduced Graphene Oxide/ITO Electrode. J. Electroanal. Chem..

[15] Bindu A. G., Bhat R. S., Manjunatha J. G., Kanthappa B. (2025). Chromium Oxide
Nanoparticles as a Modifier of Carbon Paste Electrode for Cyclic Voltametric
Assessment of Paracetamol in Medicinal Sample. Sci. Rep..

[16] G B. A., Bhat R. S. (2025). Voltammetric Investigation of Synthesized
Zirconium
Oxide Nanoparticles for Detection of Catechol. J. Environ. Chem. Eng..

[17] Shivani, Bhat R. S., Sajankila S. P., Bhat B. R. (2025). Magnesium Doped Silver Ferrite Nanohybrid for Identification
of Dihydroxybenzene Isomers. Microchem. J..

[18] Yallur B. C., Rao M. P., Harshitha M., Basrur D., Umesh P. H., Kamat V., Venu Prasad K. D., Venugopala K. N., Bhat R. S. (2025). Recent Advances in Graphene-Based
Metal Oxide Composites
for Supercapacitors: A Comprehensive Review. Adv. Sustainable Syst..

[19] Liu X., Sakthivel R., Cheng C.-Y., Luo J., Song L., Wu J., He W., Younis U., Chung R.-J. (2020). 3A-Amino-3A-Deoxy-(2AS,
3AS)-β-Cyclodextrin Hydrate/Tin Disulfide Modified Screen-Printed
Carbon Electrode for the Electrochemical Detection of Polychlorinated
Biphenyls. Nanoscale Res. Lett..

[20] Sakthivel R., He J.-H., Chung R.-J. (2022). Self-Templating
Hydrothermal Synthesis
of Carbon-Confined Double-Shelled Ni/NiO Hollow Microspheres for Diphenylamine
Detection in Fruit Samples. J. Hazard. Mater..

[21] Vinoth S., Govindasamy M., Wang S.-F., Alothman A. A., Alshgari R. A. (2021). Hydrothermally
Synthesized Cubical Zinc Manganite Nanostructure for Electrocatalytic
Detection of Sulfadiazine. Microchim. Acta.

[22] Wang N., Ma X., Xu H., Chen L., Yue J., Niu F., Yang J., Qian Y. (2014). Porous ZnMn2O4Microspheres as a Promising
Anode Material for Advanced Lithium-Ion Batteries. Nano Energy.

[23] Karuppaiah B., Anupriya J., Chen S. M., Park S. J. (2023). An Emergent
Electrochemical
Sensor Based on Spinel Zinc Manganese Oxide Decorated on Amine-Functionalized
Boron Nitride for Enhanced Electrochemical Determination of Herbicide
Mesotrione. Process Saf. Environ. Prot..

[24] Mukherjee P., Mohanty R. I., E V B., Pattnaik S., Choudhury B., Mohapatra M. (2023). Graphite-Sheathed
ZnMn2O4Microspheres for Electrochemical
Detection of As­(III). Mater. Chem. Phys..

[25] Heiba Z. K., Mohamed M. B., Badawi A. (2022). Structure
and Optical Properties
of Nano-ZnMn2O4/CuS Solid Solution Heterostructure. J. Solgel Sci. Technol..

[26] Heiba Z. K., Mohamed M. B., Badawi A. (2022). Structural
and Optical Properties
of (1 – x)­ZnMn2O4/XPbS Nanocomposites. J. Mater. Sci.:Mater. Electron..

[27] Li Y., Tang L., Deng D., Ye J., Wu Z., Wang J., Luo L. (2019). A Novel Non-Enzymatic
H2O2 Sensor
Using ZnMn2O4Microspheres Modified Glassy Carbon Electrode. Colloids Surf., B.

[28] Sakthivel R., Kubendhiran S., Chen S.-M. (2019). Facile One-Pot Sonochemical Synthesis
of Ni Doped Bismuth Sulphide for the Electrochemical Determination
of Promethazine Hydrochloride. Ultrason. Sonochem..

[29] Veerakumar P., Rajkumar C., Chen S.-M., Thirumalraj B., Lin K.-C. (2018). Activated Porous Carbon Supported
Rhenium Composites
as Electrode Materials for Electrocatalytic and Supercapacitor Applications. Electrochim. Acta.

[30] Yurkov G. Yu., Kozinkin A. V., Koksharov Y. A., Fionov A. S., Taratanov N. A., Vlasenko V. G., Pirog I. V., Shishilov O. N., Popkov O. V. (2012). Synthesis and Properties of Rhenium–Polyethylene
Nanocomposite. Composites, Part B.

[31] Dobrzańska-Danikiewicz A. D., Wolany W., Benke G., Rdzawski Z. (2014). The New MWCNTs–Rhenium
Nanocomposite. Phys. Status Solidi B.

[32] Anantharaj S., Sakthikumar K., Elangovan A., Ravi G., Karthik T., Kundu S. (2016). Ultra-Small Rhenium Nanoparticles Immobilized on DNA Scaffolds: An
Excellent Material for Surface Enhanced Raman Scattering and Catalysis
Studies. J. Colloid Interface Sci..

[33] Aboul-Gheit A. K., Abdel-Hamid S. M., Aboul-Fotouh S. M., Aboul-Gheit N. A. K. (2006). Cyclohexene
Hydroconversion Using Monometallic and Bimetallic Catalysts Supported
on Γ-Alumina. J. Chin. Chem. Soc..

[34] Veerakumar P., Thanasekaran P., Lin K.-C., Liu S.-B. (2017). Well-Dispersed Rhenium
Nanoparticles on Three-Dimensional Carbon Nanostructures: Efficient
Catalysts for the Reduction of Aromatic Nitro Compounds. J. Colloid Interface Sci..

[35] Ribeiro A. P. C., Santos B. M., Faustino R. F. C., Pombeiro A. J. L., Martins L. M. D. R. S. (2022). C-Heterogenized
Re Nanoparticles as Effective Catalysts for the Reduction
of 4-Nitrophenol and Oxidation of 1-Phenylethanol. Catalysts.

[36] Veerakumar P., Pandiyan R., Chen S.-M., Thanasekaran P., Saranya K. (2025). Recent Trends and Perspectives in Rhenium-Based Nanomaterials
for Sustainable Applications. Coord. Chem. Rev..

[37] Karthika A., Sudhakar C., Karuppasamy P., Tamilselvi B., Meena S., Anantharaju K. S., Tan K. B., Murthy H. C. A. (2024). Green
Synthesized CaO Decorated Ternary CaO/g-C3N4/PVA Nanocomposite Modified
Glassy Carbon Electrode for Enhanced Electrochemical Detection of
Caffeic Acid. Sci. Rep..

[38] Pompapathi K., Anantharaju K. S., Karuppasamy P., Subramaniam M., Uma B., Boppanahalli
Siddegowda S., Paul Chowdhury A., Murthy H. C. A. (2024). Visible-Light-Driven *Mentha Spicata* L.-Mediated Ag-Doped Bi _2_ Zr _2_ O _7_ Nanocomposite for Enhanced Degradation of
Organic Pollutants, Electrochemical
Sensing, and Antibacterial Applications. ACS
Environ. Au.

[39] Al-Balawi A. M., Zaheer Z., Kosa S. A. (2023). Facile Synthesis
of Silver-Rhenium
Nanoparticles with Plasmonic and Mesoporous Properties. J. Mol. Struct..

[40] Wang S., Zhang S., Chen X., Yuan G., Wang B., Bai J., Wang H., Wang G. (2020). Double–Shell Zinc Manganate
Hollow Microspheres Embedded in Carbon Networks as Cathode Materials
for High–Performance Aqueous Zinc–Ion Batteries. J. Colloid Interface Sci..

[41] Venkatesh K., Rajakumaran R., Chen S.-M., Karuppasamy P., Banach A., Al-Onazi W. A., Sonadevi S., Krishnan N. P., Yang C.-C., Karuppiah C., Ramaraj S. K. (2022). SrMnO3/Functionalized
h-BN Composite Modified Disposable Sensor for the Voltammetric Determination
of Furaltadone Antibiotic Drug. Catalysts.

[42] Venkatesh K., Muthukutty B., Chen S.-M., Karuppasamy P., Haidyrah A. S., Karuppiah C., Yang C.-C., Ramaraj S. K. (2022). Spinel
CoMn2O4 Nano-/Micro-Spheres Embedded RGO Nanosheets Modified Disposable
Electrode for the Highly Sensitive Electrochemical Detection of Metol. J. Ind. Eng. Chem..

[43] Ashokrao
Jagtap A., Sakthivel R., Kubendhiran S., Lin L.-Y., Kannan Ramaraj S., Liu X., Pattappan D., Hsu-Wei, Lai Y.-T., Tung C.-W., Chung R.-J. (2024). Metal-Organic Framework Derived Mn0.2Zn0.8Se/C
Amalgamated with Nitrogen-Doped Graphene Hydrogel for Antioxidant
Trolox Detection in Food, Environmental, and Biological Samples. Chem. Eng. J..

[44] Wang C., Zhou C., Zhang B., Ou X., Cao L., Peng C., Zhang J. (2019). Room-Temperature Solution Synthesis
of ZnMn _2_ O _4_ Nanoparticles for Advanced Electrochemical
Lithium Storage. RSC Adv..

[45] Saranya P. E., Selladurai S. (2018). Efficient
Electrochemical Performance of ZnMn2O4 Nanoparticles
with RGO Nanosheets for Electrodes in Supercapacitor Applications. J. Mater. Sci.:Mater. Electron..

[46] Zhang P., Li X., Zhao Q., Liu S. (2011). Synthesis and Optical Property of
One-Dimensional Spinel ZnMn2O4 Nanorods. Nanoscale
Res. Lett..

[47] Pushpanathan S., yahya S., Gunasekaran A., Natarajan S. R., Kannan K., Krishnan K. (2025). Caffeic Acid Functionalized Silver
Nanoparticles: A Bionanoformulation and Its Assessment of Cell Cycle
and in Vitro Cytotoxicity. Next Nanotechnol..

[48] Zuo F., Xie H., Gao J., Chen K., Yang H., Wang K., Meng L., Liu H. (2024). Structural Modulation of Multi-Layer
Hollow Microspheres ZnMn2O4 and Their Application in Supercapacitors. Appl. Surf. Sci..

[49] Zaheer Z., AL-Balawi A. M., Kosa S. A. (2025). Ag0, Re0, and Ag@Re
Heterogeneous
Persulfate Activators for Reactive Radical Based Oxidation of Water
Contaminant. J. Mol. Struct..

[50] Lyu L., Kim C. W., Seong K., Kang J., Liu S., Yamauchi Y., Piao Y. (2022). Defect Engineering
Induced Heterostructure
of Zn-Birnessite@spinel ZnMn2O4 Nanocrystal for Flexible Asymmetric
Supercapacitor. Chem. Eng. J..

[51] Nagaraja, P. ; Rao, H. S. ; Rao, G. R. ; Justin, P. Zinc Manganite as an Efficient Battery-Grade Material for Supercapattery Devices. May 8, 2024 10.21203/rs.3.rs-4353444/v1.

[52] Sakthivel R., Prasanna S. B., Tseng C., Lin L., Duann Y., He J., Chung R. (2022). A Sandwich-Type Electrochemical
Immunosensor for Insulin
Detection Based on Au-Adhered Cu _5_ Zn _8_ Hollow
Porous Carbon Nanocubes and AuNP Deposited Nitrogen-Doped Holey Graphene. Small.

[53] Prasanna S. B., Lin Y.-C., Ramaraj S. K., Dhawan U., Liu X., Tung C.-W., Sakthivel R., Chung R.-J. (2024). 2D/2D Heterostructure
Ni-Fe LDH/Black Phosphorus Nanosheets with AuNP for Noxious Substance
Diphenylamine Detection in Food Samples. Food
Chem..

[54] Agboola B. O., Mocheko A., Pillay J., Ozoemena K. I. (2008). Nanostructured Cobalt
Phthalocyanine Single-Walled Carbon Nanotube Platform: Electron Transport
and Electrocatalytic Activity on Epinephrine. J. Porphyrins Phthalocyanines.

[55] Bacil R. P., Garcia P. H. M., Serrano S. H. P. (2022). New
Insights on the Electrochemical
Mechanism of Epinephrine on Glassy Carbon Electrode. J. Electroanal. Chem..

[56] Mphuthi N. G., Adekunle A. S., Ebenso E. E. (2016). Electrocatalytic
Oxidation of Epinephrine
and Norepinephrine at Metal Oxide Doped Phthalocyanine/MWCNT Composite
Sensor. Sci. Rep..

[57] Lu X., Li Y., Du J., Zhou X., Xue Z., Liu X., Wang Z. (2011). A Novel Nanocomposites
Sensor for Epinephrine Detection in the Presence
of Uric Acids and Ascorbic Acids. Electrochim.
Acta.

[58] Sipuka D. S., Sebokolodi T. I., Olorundare F. O. G., Muzenda C., Nkwachukwu O. V., Nkosi D., Arotiba O. A. (2023). Electrochemical Sensing of Epinephrine
on a Carbon Nanofibers and Gold Nanoparticle-Modified Electrode. Electrocatalysis.

[59] Sakthivel R., Mutharani B., Chen S.-M., Kubendhiran S., Chen T.-W., Al-Hemaid F. M. A., Ali M. A., Elshikh M. S. (2018). A Simple
and Rapid Electrochemical Determination of L-Tryptophan Based on Functionalized
Carbon Black/Poly-L-Histidine Nanocomposite. J. Electrochem. Soc..

[60] Kumar S., Singh D., Pathania D., Awasthi A., Singh K. (2023). Molybdenum
Disulphide-Nitrogen Doped Reduced Graphene Oxide Heterostructure Based
Electrochemical Sensing of Epinephrine. Mater.
Chem. Phys..

[61] Sakthivel R., Annalakshmi M., Chen S.-M., Kubendhiran S. (2020). Synergistic
Activity of Binary Metal Sulphide WS _2_ – RuS _2_ Nanospheres for the Electrochemical Detection of the Antipsychotic
Drug Promazine. New J. Chem..

[62] Sethu
Madhavan A., Kakkaraparambil Vijayan J., Rajith L. (2022). A Layered
Electrochemical Sensor for Epinephrine Based on a Nitrogen-Doped Reduced
Graphene Oxide-ZnFe _2_ O _4_ /B-Cyclodextrin-Modified
Platinum Electrode. ChemistrySelect.

[63] Sakthivel R., Kubendhiran S., Chen S.-M., Chen T.-W., Al-Zaqri N., Alsalme A., Alharthi F. A., Abu Khanjer M. M., Tseng T.-W., Huang C.-C. (2019). Exploring the Promising Potential
of MoS2–RuS2 Binary Metal Sulphide towards the Electrocatalysis
of Antibiotic Drug Sulphadiazine. Anal. Chim.
Acta.

[64] Elugoke S. E., Fayemi O. E., Adekunle A. S., Sherif E.-S. M., Ebenso E. E. (2022). Electrochemical
Sensor for the Detection of Adrenaline at Poly­(Crystal Violet) Modified
Electrode: Optimization and Voltammetric Studies. Heliyon.

[65] Mutić T., Ognjanović M., Kodranov I., Robić M., Savić S., Krehula S., Stanković D. M. (2023). The Influence
of Bismuth Participation on the Morphological and Electrochemical
Characteristics of Gallium Oxide for the Detection of Adrenaline. Anal. Bioanal. Chem..

[66] Fouad D. M., El-Said W. A. (2016). Selective Electrochemical
Detection of Epinephrine
Using Gold Nanoporous Film. J. Nanomater..

[67] Sakthivel R., Liu T.-Y., Chung R.-J. (2023). Bimetallic
Cu5Zn8 Alloy-Embedded
Hollow Porous Carbon Nanocubes Derived from 3D-Cu/ZIF-8 as Efficient
Electrocatalysts for Environmental Pollutant Detection in Water Bodies. Environ. Res..

[68] Elugoke S. E., Fayemi O. E., Adekunle A. S., Ganesh P.-S., Kim S.-Y., Ebenso E. E. (2023). Sensitive and Selective Neurotransmitter
Epinephrine
Detection at a Carbon Quantum Dots/Copper Oxide Nanocomposite. J. Electroanal. Chem..

[69] da
Silva L. V., Lopes C. B., da Silva W. C., de Paiva Y. G., dos Santos Silva F. D. A., Lima P. R., Kubota L. T., Goulart M. O. F. (2017). Electropolymerization of Ferulic Acid on Multi-Walled
Carbon Nanotubes Modified Glassy Carbon Electrode as a Versatile Platform
for NADH, Dopamine and Epinephrine Separate Detection. Microchem. J..

[70] Yao H., Sun Y., Lin X., Tang Y., Liu A., Li G., Li W., Zhang S. (2007). Selective Determination of Epinephrine in the Presence
of Ascorbic Acid and Uric Acid by Electrocatalytic Oxidation at Poly­(Eriochrome
Black T) Film-Modified Glassy Carbon Electrode. Anal. Sci..

[71] Ren W., Luo H. Q., Li N. B. (2006). Simultaneous Voltammetric Measurement
of Ascorbic Acid, Epinephrine and Uric Acid at a Glassy Carbon Electrode
Modified with Caffeic Acid. Biosens. Bioelectron..

[72] Pecheu C., Tchieda V., Tajeu K., Jiokeng S., Lesch A., Tonle I., Ngameni E., Janiak C. (2023). Electrochemical Determination
of Epinephrine in Pharmaceutical Preparation Using Laponite Clay-Modified
Graphene Inkjet-Printed Electrode. Molecules.

[73] Roychoudhury A., Prateek A., Basu S., Jha S. K. (2018). Preparation and
Characterization of Reduced Graphene Oxide Supported Nickel Oxide
Nanoparticle-Based Platform for Sensor Applications. J. Nanopart. Res..

[74] Ramu A. G., Umar A., Ibrahim A. A., Algadi H., Ibrahim Y. S. A., Wang Y., Hanafiah M. M., Shanmugam P., Choi D. (2021). Synthesis of Porous 2D Layered Nickel
Oxide-Reduced Graphene Oxide
(NiO-RGO) Hybrid Composite for the Efficient Electrochemical Detection
of Epinephrine in Biological Fluid. Environ.
Res..

