# Supplementary material for: Caffeic Acid-Driven Green Synthesis of Rhenium Nanoparticles Embedded in Self-Templating Double-Shelled ZnMn2O4 Hollow Microspheres for Ultrasensitive Epinephrine Detection in Biofluids
Source: ACS Sens. 2025 Nov 27;11(1):324–37. doi: 10.1021/acssensors.5c03023 (PMC12836355; doi:10.1021/acssensors.5c03023)
Supplement: Supplementary file 1 [file se5c03023_si_001.pdf]

Supporting Information

Caffeic Acid-Driven Green Synthesis of Rhenium Nanoparticles Embedded in Self-Templating Double-Shelled $ZnMn_2O_4$ Hollow Microspheres for Ultrasensitive Epinephrine Detection in Biofluids

Rajalakshmi Sakthivel [1,*], Bo-Yuan Chen [1], Subbiramaniyan Kubendhiran [1], Lu-Yin Lin [1], Sayee Kannan Ramaraj [2], Yu-Chien Lin [3,4], Xinke Liu [5,6], Cihun-Siyong Gong [7,*], Ren-Jei Chung [1,8,*]

[1] Department of Chemical Engineering and Biotechnology, National Taipei University of Technology (Taipei Tech), Taipei 10608, Taiwan

[2] PG& Research Department of Chemistry, Thiagarajar College, Madurai 625009, Tamil Nadu, India

[3] School of Materials Science and Engineering, Nanyang Technological University, Singapore 639798, Singapore

[4] BIOBOND LTD, Corsham, Wiltshire SN13 0B, United Kingdom

[5] College of Materials Science and Engineering, Chinese Engineering and Research Institute of Microelectronics, Shenzhen University, Shenzhen 518060, China

[6] Department of Electrical and Computer Engineering, National University of Singapore, Singapore 117583, Singapore

[7] Department of Electrical Engineering, National Central University, Zhongli, Taoyuan 320317, Taiwan

[8] High-value Biomaterials Research and Commercialization Center, National Taipei University of Technology (Taipei Tech), Taipei 10608, Taiwan

* Corresponding Author: Prof. Ren-Jei Chung

Email: rjchung@mail.ntut.edu.tw; Tel: (886-2) 2771-2171 ext 2547

Also corresponding to Prof. Rajalakshmi Sakthivel (rajalakshmi@ntut.edu.tw) and Prof. Cihun-Siyong Gong (alexgong@cc.ncu.edu.tw)

Address: Department of Chemical Engineering and Biotechnology, National Taipei University of Technology (Taipei Tech), No. 1, Sec. 3, Zhongxiao E. Rd., Taipei 10608 Taiwan

<u>Contents</u>

S1. Chemicals and reagents

S2. Characterization techniques

Number of Figures: 08

Number of Tables: 01

Number of Pages: 09

S1. Chemicals and reagents

(−)-Epinephrine (EP, ≥99%), ammonium perrhenate (NH_4ReO_4, 99.999%), sodium hydroxide (NaOH, ≥97.0%), hydrochloric acid (HCl, 37%), caffeic acid (CA, >=98.0%), hexamethylenetetramine (HMTA, ≥99.0%), sodium tartrate dihydrate ($C_4H_4O_6Na_2{\cdot}2H_2O$, 99.0%) potassium hexacyanoferrate (II) ($K_4[Fe(CN)_6]$, 98.5-102.0%), potassium hexacyanoferrate(III) ($K_3[Fe(CN)_6]$, ≥99.0%), ascorbic acid (AA, ≥99.0%), corticosterone (CORT, ≥98.5%), dopamine hydrochloride (DA, ≥98%), 17α-Ethinylestradiol (EE2, ≥98%), β-Estradiol (EST, ≥98%), glucose (GLU, ≥99.5%), hydrogen peroxide (H_2O_2, ≥30%), indomethacin (INDO, ≥98%), mefenamic acid (MFA, > 98%), (−)-Norepinephrine (NEP, ≥98%), acetaminophen (PA, ≥99.0%), and serotonin (SER, 98%), were purchased from Sigma Aldrich (USA). Manganese(II) sulfate tetrahydrate ($MnSO_4{\cdot}4H_2O$, 99%), zinc sulfate heptahydrate ($ZnSO_4{\cdot}7H_2O$, 99.0-103.0%), and cetyltrimethylammonium bromide (CTAB, 98%) were purchased from Alfa Aesar (USA). Sodium chloride (NaCl, 99.5%) and ethanol (EtOH, 99.8%) were purchased from Honeywell (USA). Potassium chloride (KCl, 100.5%), potassium dihydrogen phosphate (H_2KO_4P, 99%), and sodium dihydrogen phosphate ($NaH_2PO_4{\cdot}2H_2O$, 98.0-103.0%) were purchased from J.T. Baker (USA). The pH of the phosphate buffer was adjusted using 0.1 M NaOH and 1 N HCl solutions. All the materials were of analytical grade.

S2. Characterization techniques

The morphological features of the synthesized samples were examined using field-emission scanning electron microscopy (FE-SEM, Regulus-8100, Hitachi, USA) and transmission electron microscopy (TEM, JEM 2100 F; JEOL Ltd., Tokyo, Japan). The elemental composition was determined using energy-dispersive X-ray analysis (EDX), and the crystalline structure and phase purity were assessed using X-ray powder diffraction (XRD; X'Pert3 Powder, Malvern Panalytical, UK). The functional groups and chemical bonds were qualitatively analyzed using Fourier-transform infrared spectroscopy (FTIR, Spotlight 200i Sp2 with AutoATR System, Perkin Elmer, USA) and X-ray photoelectron spectroscopy (XPS, JEOL Ltd., JPS-9030). The electrochemical experiments were conducted on a CHI6111E workstation (CH Instruments, USA) using a three-electrode system. A glassy carbon electrode (SPCE, 0.071 cm^2 working area) acted as the working electrode. At the same time, saturated silver/silver chloride (Ag/AgCl) and a platinum (Pt) filament functioned as the reference and counter electrodes, respectively. The

electrochemical characteristics of the materials were assessed using cyclic voltammetry (CV), electrochemical impedance spectroscopy (EIS), and differential pulse voltammetry (DPV). Increasing concentrations of EP were detected using the DPV technique, with a potential window of -0.2 to 0.8 V, a pulse width of 0.05 s, and a sensitivity (A/V) of 1×10^{-5}.

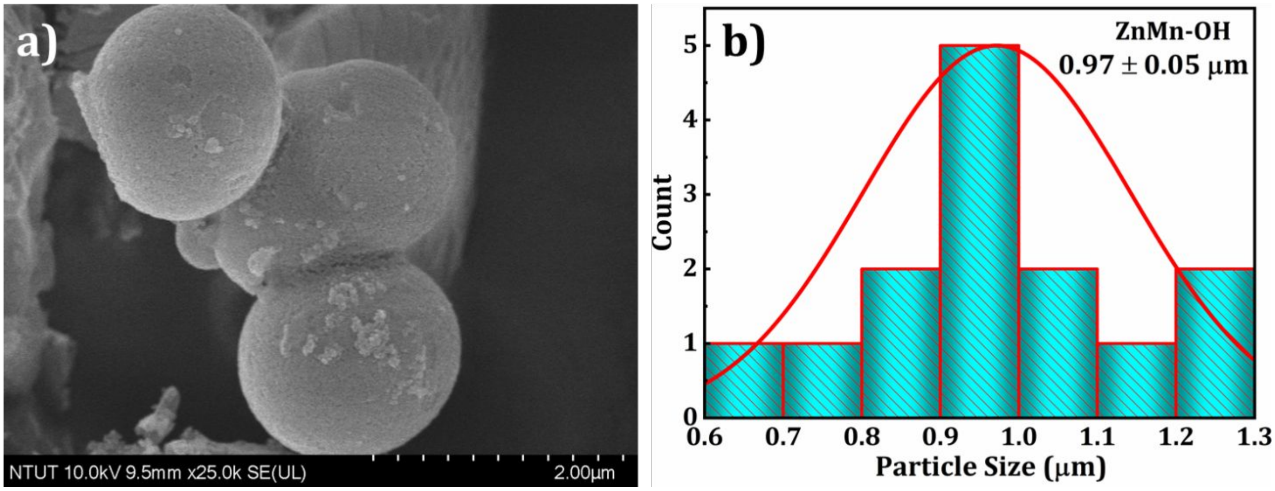

Figure S1. (a) FESEM image and (b) particle size distribution diagram of ZnMn-hydroxide.

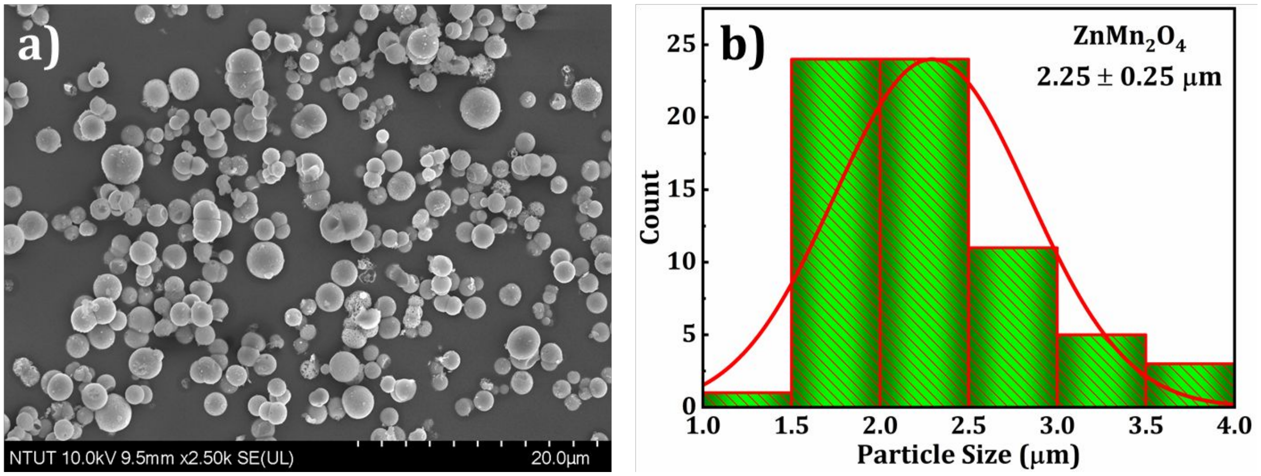

Figure S2. (a) FESEM image and (b) particle size distribution diagram of $ZnMn_2O_4$.

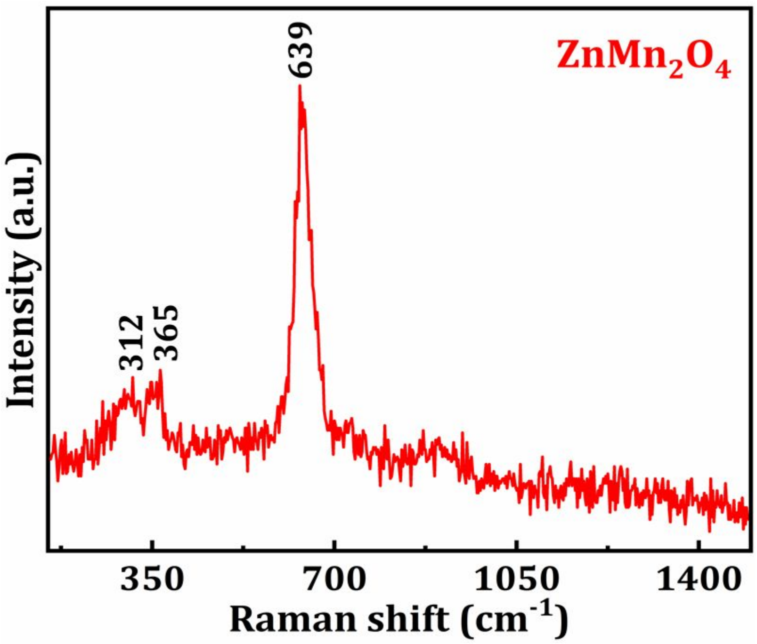

Figure S3. Raman spectrum of $ZnMn_2O_4$.

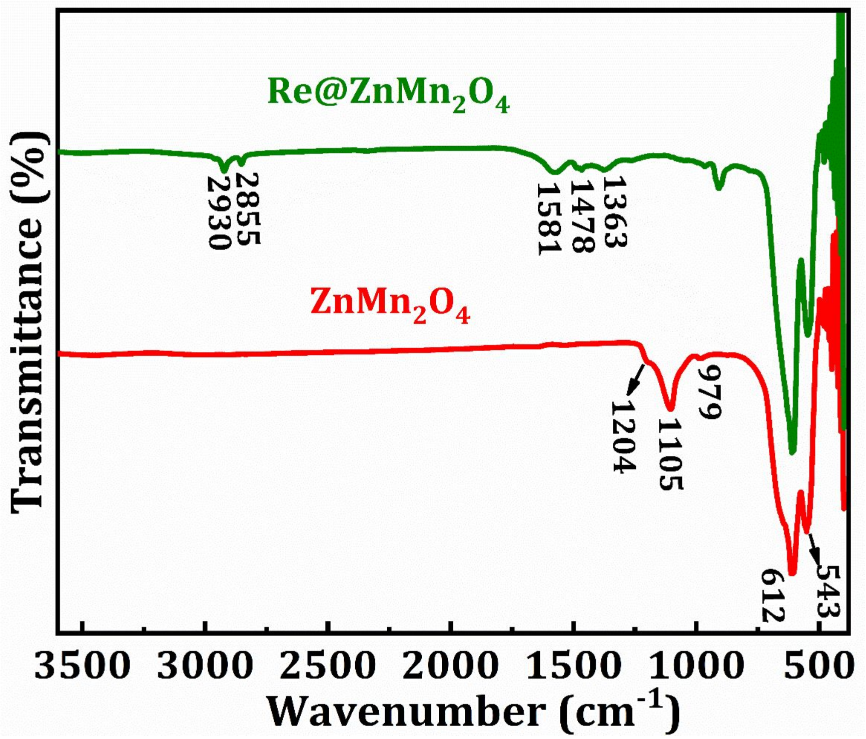

Figure S4. FT-IR spectrum of $ZnMn_2O_4$ and Re@$ZnMn_2O_4$.

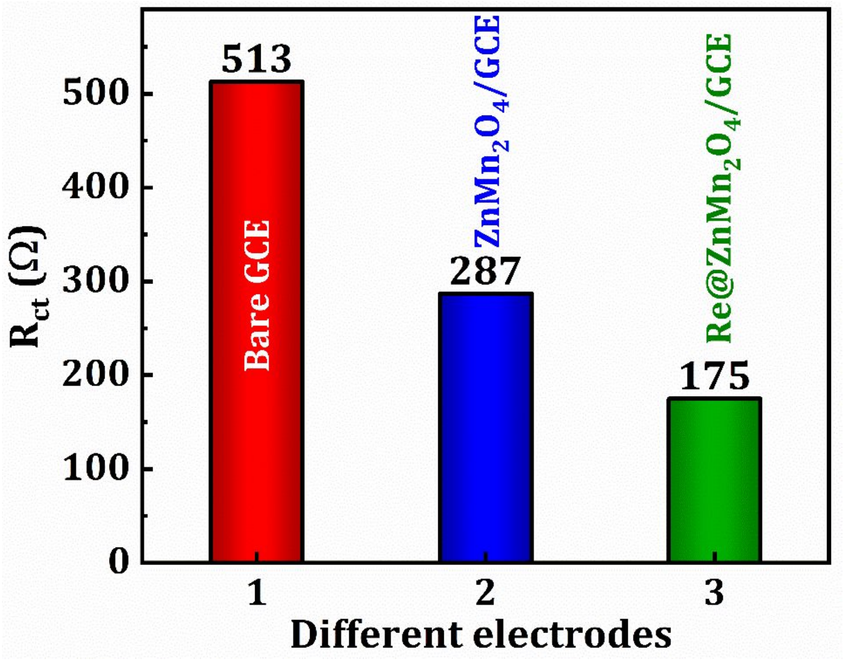

Figure S5. Bar chart of different electrodes and their charge transfer resistance (R_{ct}) values.

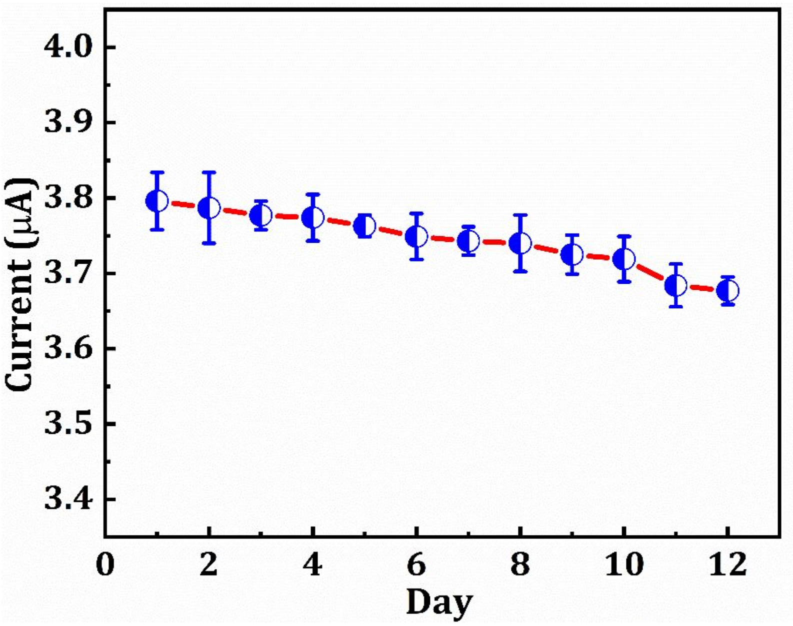

Figure S6. The plot for the storage stability of Re@$ZnMn_2O_4$/GCE over 12 days under a storage condition of 4 °C.

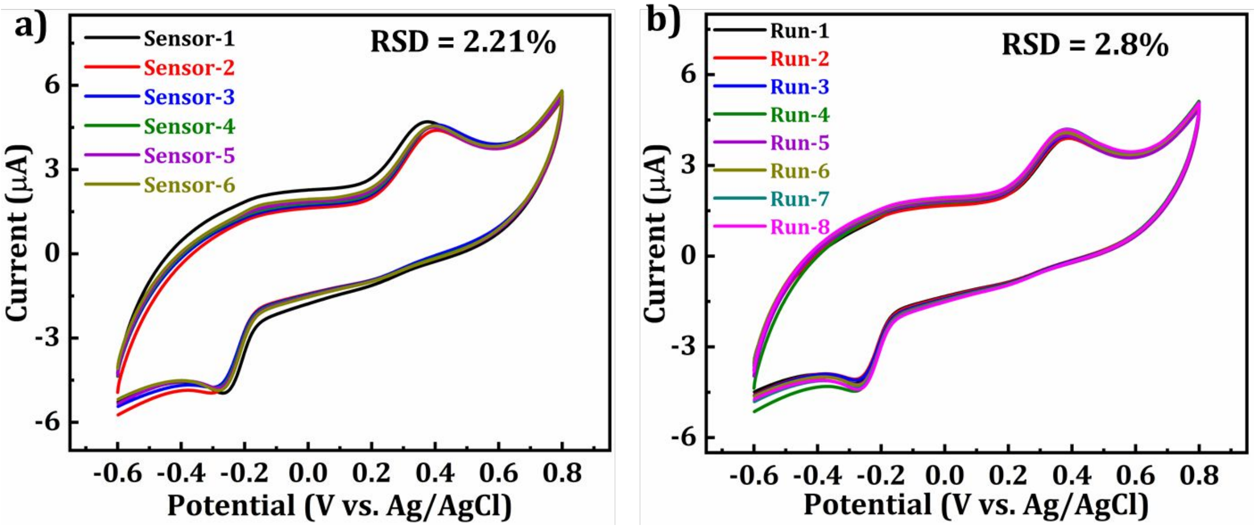

Figure S7. CV curves of the (a) reproducibility and (b) repeatability analysis at the Re@$ZnMn_2O_4$/GCE in 50 μM EP containing 0.1 M PBS (pH=7.0).

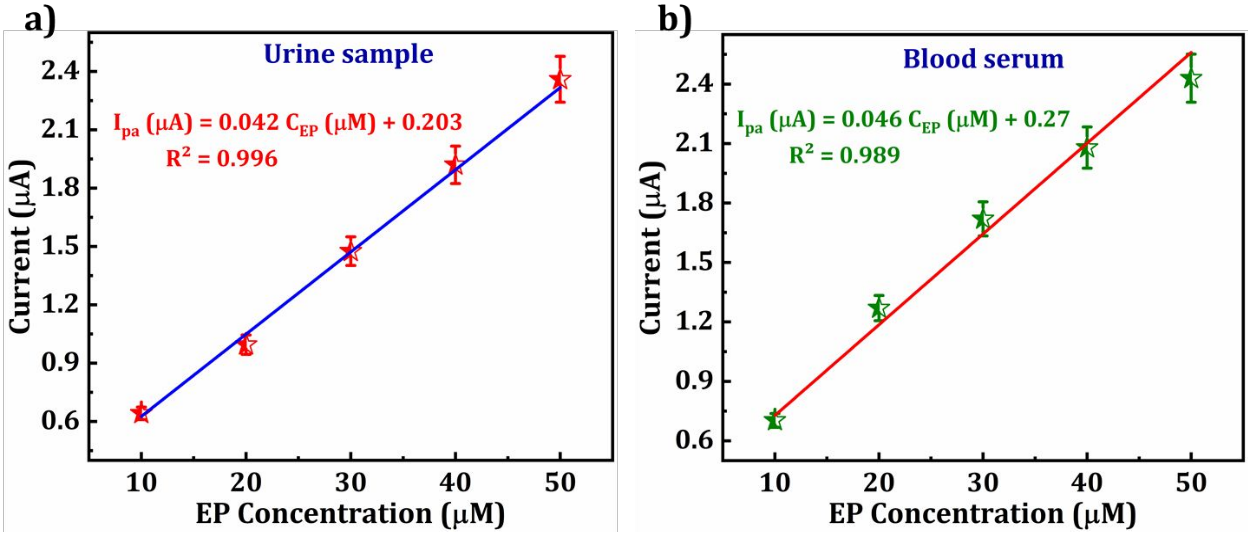

Figure S8. (a & b) Linear plots for the real sample analysis of urine and blood serum samples.

Table S1. Determination of EP in human urine and blood serum samples using the Re@$ZnMn_2O_4$/GCE sensor (n=3).

Human samples	Added (µM)	Found (µM)	Recovery (%)	RSD (%)
Urine	0	-	-	-
	10	9.95	99.50	2.05
	20	19.85	99.25	1.82
	30	29.28	97.60	2.36
	40	38.90	97.25	3.17
	50	49.55	99.10	2.19
Blood serum	0	-	-	-
	10	9.90	99.00	1.98
	20	19.89	99.45	2.24
	30	28.98	96.60	2.07
	40	39.63	99.08	1.52
	50	49.76	99.52	2.15
